# Supplementary material for: Incidence of anorexia nervosa in young people in the UK and Ireland: a national surveillance study
Source: BMJ Open. 2019 Oct 22;9(10):e027339. doi: 10.1136/bmjopen-2018-027339 (PMC6954494; doi:10.1136/bmjopen-2018-027339)
Supplement: Author's manuscript [file bmjopen-2018-027339.draft_revisions.pdf]

BMJ Open

BMJ Open is committed to open peer review. As part of this commitment we make the peer review history of every article we publish publicly available.

When an article is published we post the peer reviewers' comments and the authors' responses online. We also post the versions of the paper that were used during peer review. These are the versions that the peer review comments apply to.

The versions of the paper that follow are the versions that were submitted during the peer review process. They are not the versions of record or the final published versions. They should not be cited or distributed as the published version of this manuscript.

BMJ Open is an open access journal and the full, final, typeset and author-corrected version of record of the manuscript is available on our site with no access controls, subscription charges or pay-per-view fees (http://bmjopen.bmj.com).

If you have any questions on BMJ Open's open peer review process please email info.bmjopen@bmj.com

BMJ Open

Incidence of anorexia nervosa in young people in the United Kingdom and the Republic of Ireland: A national surveillance study

Journal:	*BMJ Open*
Manuscript ID	bmjopen-2018-027339
Article Type:	Research
Date Submitted by the Author:	17-Oct-2018

Complete List of Authors:	Petkova, Hristina; King's College London, Health Service and Population Research Department<br>Simic, Mima; South London and Maudsley NHS Foundation Trust<br>Nicholls, Dasha; Imperial College London<br>Ford, Tamsin; University of Exeter, Medical School<br>Prina, A.Matthew; King's College London,<br>Stuart, Ruth; King's College London, Health Service and Population Research Department<br>Livingstone, Nuala; Queen's University Belfast<br>Kelly, Grace; Queen's University Belfast<br>Macdonald, Geraldine; University of Bristol<br>Eisler, Ivan; South London and Maudsley NHS Foundation Trust<br>Gowers, Simon; University of Liverpool<br>Barrett, Barbara; King's College London<br>Byford, Sarah; King's College London, Health Service and Population Research Department

Keywords:	Eating disorders < PSYCHIATRY, Child & adolescent psychiatry < PSYCHIATRY, EPIDEMIOLOGY

SCHOLARONE™
Manuscripts

Incidence of anorexia nervosa in young people in the United Kingdom and the Republic of Ireland: A national surveillance study

Hristina Petkova, Institute of Psychiatry, Psychology & Neuroscience at King's College London (Research Fellow).

Mima Simic, South London and Maudsley NHS Foundation Trust (Consultant Child and Adolescent Psychiatrist).

Dasha Nicholls, Imperial College London (Reader in Child and Adolescent Psychiatry).

Tamsin Ford, University of Exeter Medical School (Professor of Child and Adolescent Psychiatry).

A. Matthew Prina, Institute of Psychiatry, Psychology & Neuroscience at King's College London (Senior Lecturer in Epidemiology).

Ruth Stuart, Institute of Psychiatry, Psychology & Neuroscience at King's College London (Research Assistant).

Nuala Livingstone, Queen's University Belfast (Research Fellow).

Grace Kelly, Queen's University Belfast (Research Fellow).

Geraldine Macdonald, University of Bristol (Professor of Social Work).

Ivan Eisler, South London and Maudsley NHS Foundation Trust (Professor of Family Psychology and Family Therapy).

Simon Gowers, University of Liverpool (Professor of Adolescent Psychiatry).

Barbara Barrett, Institute of Psychiatry, Psychology & Neuroscience at King's College London (Senior Lecturer in Health Economics).

Sarah Byford, Institute of Psychiatry, Psychology & Neuroscience at King's College London (Professor of Health Economics).

Corresponding author: Sarah Byford, Institute of Psychiatry, Psychology & Neuroscience at King's College London, P024 David Goldberg Centre, De Crespigny Park, London SE5 8AF, UK, s.byford@kcl.ac.uk, 02078480043

For peer review only - http://bmjopen.bmj.com/site/about/guidelines.xhtml

For peer review only

Abstract

Objectives: This study aimed to estimate the incidence of DSM5 anorexia nervosa in young people in contact with child and adolescent mental health services in the UK and Republic of Ireland.

Design: Observational, surveillance study using the Child and Adolescent Psychiatry Surveillance System, involving monthly reporting by child and adolescent psychiatrists between 1st February 2015 and 30th September 2015.

Setting: UK and Republic of Ireland

Participants: Clinician-reported data on young people aged 8 to 17 in contact with child and adolescent mental health services for a first episode of anorexia nervosa.

Main outcome measures: Annual incidence rates estimated as confirmed new cases per 100,000 population at risk.

Results: 305 incident cases of anorexia nervosa were reported over the 8-month surveillance period and assessed as eligible for inclusion. The majority were young women (91%), from England (70%), and of white ethnicity (92%). Mean age was 14.6 years (±1.66) and mean percentage of median expected BMI for age and sex was 83.23% (±10.99%). The overall incidence rate, adjusted for missing data, was estimated to be 13.68 per 100,000 population (95% CI 12.88 to 14.52), with rates of 25.66 for young women (95% CI 24.09 to 27.30) and 2.28 for young men (95% CI 1.84 to 2.79). Incidence increased steadily with age, peaking at 15 for young women (57.77, 95% CI 50.41 to 65.90) and 16 for young men (5.14, 95% CI 3.20 to 7.83). Comparison with earlier estimates suggests incidence rates for children aged 13 and under have increased over the last ten years.

Conclusions: These results provide up-to-date estimates of the incidence of anorexia nervosa in young people. Service providers and commissioners should consider evidence to suggest an increase in incidence in younger children.

Study registration: International Standard Randomised Controlled Trial Number Register [ISRCTN12676087].

For peer review only - http://bmjopen.bmj.com/site/about/guidelines.xhtml

Strengths and limitations of this study

- Estimates of the incidence of anorexia nervosa in the UK and Republic of Ireland are limited and at least ten years old.
- This study provides up-to-date estimates of the incidence of DSM5 anorexia nervosa in young people aged 8 to 17 years in contact with child and adolescent mental health services across the UK and Republic of Ireland.
- The surveillance design of this observational study ensured a large, nationally representative sample.
- Missing data due to non-response was a limitation and required adjustments to observed data to account for the impact of this missing data.

For peer review only

For peer review only - http://bmjopen.bmj.com/site/about/guidelines.xhtml

Introduction

Anorexia nervosa is a serious and enduring eating disorder with high morbidity and the highest mortality among psychiatric disorders.[1] Young women are particularly susceptible, with annual UK estimates of 37 new diagnoses of anorexia nervosa per 100,000 for girls aged 10 to 19 years, compared to 3 per 100,000 for boys of the same age.[2] Prevalence estimates in young people range from 0.3% to 0.6%.[3–4]

Accurate epidemiological estimates of the number of new anorexia nervosa cases per year and their sex and age profile are needed for causal investigations and service planning.[2] However, available estimates in the UK are at least ten years old.[2,5–7] In addition, most estimates are derived from community-based primary care records,[2,5] which fail to accurately record all new cases.[8,9] Undetected anorexia nervosa cases may present to accident and emergency and require immediate paediatric or psychiatric input, including inpatient admission. Some young people may therefore bypass primary care and, consistent with UK guidelines,[10] are likely to be assessed and diagnosed by a child and adolescent psychiatrist in a secondary care setting, making secondary care records a more reliable source of data on anorexia nervosa incidence than primary care registers.

Existing incidence data from secondary care settings in the UK are limited. One study, focusing only on adolescents aged 13 to 18, was limited to Greater London and reported presentation rates to child and adolescent mental health services (CAMHS), rather than incidence estimates.[6] A second study, which used a national surveillance design, focused only on children under 13.[7] The current study aimed to estimate the incidence of anorexia nervosa in secondary care services for young people between the ages of 8 and 17 years in the UK and the Republic of Ireland. This work formed part of a study exploring the cost-effectiveness of models of care for young people with eating disorders (the CostED study).[11]

Methods

Design

An observational, surveillance study was undertaken using the Child and Adolescent Psychiatry Surveillance System (CAPSS). CAPSS is a system designed to ascertain cases of rare childhood

For peer review only - http://bmjopen.bmj.com/site/about/guidelines.xhtml

mental health conditions in the UK and Republic of Ireland through monthly reporting by clinicians and relies on non-consent to maximise the accuracy of epidemiological estimates. The CAPSS system has been operating since 2009[12] and is based on the well-established British Paediatric Surveillance Unit (BPSU) system.[13]

Ethics approvals

The study was approved by the CAPSS Executive Committee, King's College London Research Ethics Committee [PNM/13/14-105], and the Health Research Authority Confidentiality Advisory Group [CAG 4-03(PR1)/2014] under Section 251 of the NHS Act 2006, which enables disclosure of confidential patient information for purposes where it is not possible to use anonymised information and where seeking consent is not practical.

Patient and public involvement

The CostED study included a patient and a parent representative on the study steering committee who contributed to the design, conduct and management of the study, including the incidence component. A lay summary of the CostED study results will be produced, in collaboration with Beat, the national eating disorder charity, for dissemination via the Beat website.

Inclusion and exclusion criteria

The study included young people between 8 and 17 years of age, in contact with CAMHS for a first episode of anorexia nervosa according to DSM5 diagnostic criteria.[14] New cases were notified for a period of eight months from 1st February to 30th September 2015. Cases whose clinician-reported data were insufficient to assess eligibility were excluded, as were duplicate cases notified more than once by the same or different clinicians.

Procedures

At the time of the study, CAPSS used a report card, known as the yellow card, containing a list of conditions being surveyed. Yellow cards, along with reporting instructions and protocols for new studies, are sent monthly from the CAPSS office to a mailing list of all hospital, university and community child and adolescent consultant psychiatrists across the United Kingdom and the Republic of Ireland. Reporting clinicians are asked to check boxes against any of the reportable

For peer review only - http://bmjopen.bmj.com/site/about/guidelines.xhtml

conditions they have seen in the preceding month, or to check a "nil return" box and return the card to CAPSS. A tear-off slip is provided for respondents to keep a record of the patients reported. "Positive" returns are allocated a unique CAPSS ID number and notified to the appropriate research investigator, who then contacts the reporting clinician directly to request completion of a questionnaire using the CAPSS ID to enable the clinician to identify the relevant patient.

For the CostED study, the yellow card contained a check box for anorexia nervosa and was sent to clinicians along with a protocol card detailing the case notification definition for anorexia nervosa. The case notification definition (see web extras) was based on DSM5 diagnostic criteria for anorexia nervosa and was intended to aid clinicians in their decision to tick “yes” or “no” on the yellow card. It was not intended to identify whether a case met study inclusion criteria, which was determined by the research group after receipt of all necessary data.

Data

Questionnaires sent to clinicians reporting a positive case of anorexia nervosa, identified via the unique CAPSS ID number, were completed from clinical records and clinicians requested to provide data relating to the time the case was initially assessed and diagnosed. The questionnaire covered clinical features to enable assessment of case eligibility, referral pathway information to ensure assessment and diagnosis had not happened prior to the study surveillance period, and a limited set of standard patient identifiers in line with CAPSS procedures and ethics requirements, which were used to describe the sample and to identify duplicate notifications. In addition, clinicians were asked to confirm whether the case was a first episode of anorexia nervosa that had come to the attention of services.

The patient identifiers included NHS or Community Health Index (CHI) number (unique patient identifiers used in the regions of interest), hospital number, first half of postcode or town of residence for the Republic of Ireland, sex, date of birth and ethnicity (White, Mixed, Asian, Black, Chinese, Other or Unknown). In Northern Ireland, identifiers were limited to age in years and months and hospital identifier rather than hospital number, to reduce the risk of patient identification given the small geographic area. All patient identifiable data from Northern Ireland

For peer review only - http://bmjopen.bmj.com/site/about/guidelines.xhtml

were retained by the local research team, de-duplicated, anonymised and subsequently sent to the central research team in King's College London for analysis as per requirements set out by the Northern Ireland Privacy Advisory Committee. All data storage was compliant with the EU General Data Protection Regulations.

Clinical features included: weight and height to calculate body mass index (BMI) and percentage of median expected BMI for age and sex interpreted around the 85% threshold;[15] The Health of the Nation Outcome Scales for Children and Adolescents (HoNOSCA[16]), a routine outcome measure rating 13 clinical features on a five-point severity scale including behaviours, impairments, symptoms, and social functioning of children and adolescents with mental health problems; the clinician completed Children's Global Assessment Scale (CGAS[17]) used to rate emotional and behavioural functioning of young people; and a range of symptoms relating to the diagnosis of anorexia nervosa.

Unreturned or incomplete questionnaires were chased via email and telephone. Cases where any symptom required for case definition was absent despite chasing, were assessed for eligibility by a consultant child and adolescent psychiatrist (MS).

Case eligibility

Cases were assessed as eligible for the study if: (a) they were between 8 and 17 years of age; (b) they had no previous episode of anorexia nervosa that had come to the attention of services; (c) they received a clinical assessment in the reporting service during the study surveillance period; (d) they had not been referred from another secondary health service (to ensure assessment and diagnosis had not happened prior to study surveillance period); and (e) the following clinical symptoms were present: "Restriction of energy intake relative to requirements" and "Persistent behaviour that interferes with weight gain, despite low weight". This broad definition was subsequently checked using a tighter DSM5 analytic definition including the following symptoms:

1) "Restriction of energy intake relative to requirements" and
2) "Intense fear of gaining weight or of becoming fat" or "Persistent behaviour that interferes with weight gain, despite low weight" and

For peer review only - http://bmjopen.bmj.com/site/about/guidelines.xhtml

3) “Perception that body shape/size is larger than it is” or “Preoccupation with body weight and shape” or “Lack of recognition of the seriousness of the current low body weight”

Only one case which met the broad criteria failed to meet the tighter criteria, thus confirming the validity of the broad criteria.

Removal of duplicates

Duplicates were identified by comparing NHS/CHI numbers, hospital numbers/ hospital identifiers and date of birth/age in years and months, as appropriate. The management of duplicates depended upon the outcome for the original notification for which a duplicate was identified. Four scenarios were considered: (1) duplicates where the original notification met study inclusion criteria were excluded and the original retained; (2) duplicates where the original notification had been excluded because the young person was under 8 years of age or did not meet the clinical criteria were assessed as a new case to determine if the case now met eligibility criteria; (3) duplicates where the original notification was excluded due to a previous episode of anorexia nervosa, a diagnosis date prior to the study surveillance period or referral from another secondary care service, were excluded; and (4) duplicates where the original notification contained insufficient information to judge eligibility were checked to see if the duplicate contained the missing information and, if available, the original notification was reassessed for eligibility and the duplicate managed as per the scenarios above.

Data analysis

Data analysis was performed using Stata IC v14.2 and Microsoft Excel 2010. Observed incidence rates (denoted IR0), defined as the number of new cases during a specified period of time in a population at risk for developing the disease, were calculated as follows: the number of confirmed new cases of anorexia nervosa in the 8-month surveillance period converted to 12 months [(N cases over 8 months/8)*12], divided by the population at risk and multiplied by 100,000 to give the rate per 100,000 young people.

*IR0 = (confirmed new cases converted to 12 months)/the population at risk *100,000*

For peer review only - http://bmjopen.bmj.com/site/about/guidelines.xhtml

The population at risk was calculated as the total number of children of each year of age and each sex in the UK and Republic of Ireland minus the number of prevalent cases who, once diagnosed, are no longer part of the "at risk" population. Population data for 2015 were obtained from the Office for National Statistics for the UK[18] and the Central Statistics Office for the Republic of Ireland.[19] To estimate the number of prevalent cases each year, incident cases in the previous age band were used as a proxy. For example, incident cases aged eight were used as a proxy for prevalent cases in the estimation of the 'at risk' population aged nine, and so on.

To consider incidence among unobserved missing cases, adjustments were needed for unreturned CAPSS notification cards and questionnaires. For CAPSS notification cards, just over half of all notification cards sent out were returned (50.16%). To account for incidence among the 49.84% of unreturned cards, two assumptions were made, and an appropriate correction applied to IR0, the observed incidence rate:

Assumption 1: To take into consideration the possibility that unreturned cards are more likely to be 'nil' returns, it was assumed that half of unreturned cards (24.92%) were 'negative' and half followed the same proportion of 'negative' and 'positive' as the returned cards. This assumption translates into a correction coefficient of 1.50 derived from (24.92+50.16)/50.16.

Assumption 2: Making no assumptions of bias in the likelihood of unreturned cards being either positive or negative returns, it was assumed that all unreturned cards followed the same proportion of 'negative' and 'positive' as returned cards. This assumption translates into a correction coefficient of 1.99 derived from (49.84+50.16)/50.16.

These assumptions provide a range of incidence rates, from a minimum (observed incidence rate) to a maximum (assumption 2), within which the actual rate is likely to fall. We hypothesised that assumption 1 provides the most realistic estimate since it assumes a bias in the response rates with greater likelihood that unreturned cards are negative ('nil' returns) but does not assume *all* unreturned cards are 'nil' returns, which is the implicit assumption within IR0.

For peer review only - http://bmjopen.bmj.com/site/about/guidelines.xhtml

For unreturned questionnaires, approximately two-thirds of the questionnaires that were sent to clinicians reporting positive cases of anorexia nervosa were returned (63%), leaving one-third (37%) unreturned. Since all these questionnaires relate to a 'positive' notification, we applied a correction coefficient of 1.59 derived from (37+63)/63, which assumes that the incidence rate for the unreturned questionnaires is the same as the incidence rate identified in the returned questionnaires for each year of age.

We then combined the correction coefficients described above, to generate two adjusted incidence rates:

Adjusted incidence rate 1 (IR1) = Confirmed new cases of anorexia nervosa converted to 12 months, multiplied by the correction for unreturned CAPSS notification cards under assumption 1, multiplied by the correction for unreturned questionnaires, then divided by the population at risk and multiplied by 100,000**.**

*IR1 = (confirmed new cases converted to 12 months * 1.50 * 1.59)/the population at risk *100,000*

Adjusted Incidence rate 2 (IR2) = Confirmed new cases of anorexia nervosa converted to 12-months, multiplied by the correction for unreturned CAPSS notification cards under assumption 2, multiplied by the correction for unreturned questionnaires and then divided by the population at risk and multiplied by 100,000.

*IR2 = (confirmed new cases converted to 12 months * 1.99 * 1.59)/the population at risk * 100,000*

For each incidence rate, IR0, IR1 and IR2, total, age-specific and sex-specific annual incidence rates for anorexia nervosa for the year 2015 and 95% confidence intervals were calculated based on the Poisson distribution[20] using the Stata command *ci means [N new anorexia nervosa cases 12m], Poisson [exposure(total population)]* for positive integers/whole incidence numbers (Stata

For peer review only - http://bmjopen.bmj.com/site/about/guidelines.xhtml

interprets any non-integer decimal point number between 0 and 1 as the fraction of events and converts it to an integer number). Annual incidence rates were stratified by discrete age and sex.

Results

Case ascertainment

Case ascertainment is outlined in Figure 1. Over the eight-month surveillance period, 6401 yellow cards were sent to reporting clinicians and 3211 were returned (50%). Of these, 997 positive cases of anorexia nervosa were reported and 2214 were nil returns. Of the positve cases, 48 (5%) were excluded due to clinicans stating that they did not wish to be included in the study (due to retirement, shortage of reporting capacity etc.) or due to reporting errors. Questionnaires were sent to the remaining 949, and a further 352 positive cases (37%) were excluded as they failed to return the questionnaires, so no data were available to assess eligibility. Questionnaires were completed and returned for 597 notified cases, of which 292 (49%) were ineligible for reasons related to age, previous episode of anorexia nervosa, date of assessment outside the study's surveillance period, referral from another secondary care service, insufficient information to assess diagnosis or duplicate notifications, leaving 305 incident cases of anorexia nervosa as the sample for analysis.

Demographics and clinical features

Of the 305 young people identified as having DSM5 anorexia nervosa, the majority were girls (91%), from England (70%) and of white ethnicity (92%) (see Table 1). The mean age was 14.6 years (±1.66). Clinical variables suggest these young people were significantly impaired. Mean BMI was 16.50 kg/m^2 (±2.25), where values of 16.00 to 16.99 suggesting moderate severity of anorexia nervosa. Mean percentage of median expected BMI for age and sex (the deviation from expected body weight) was 83.23% (±10.99%), falling within the range required for a diagnosis of anorexia nervosa (<85%). Mean CGAS score was 44.61 (±14.08), which falls within the range for 'obvious problems' (41–50) on a scale from 1 to 100 (1 being the worst and 100 the best emotional and behavioural functioning). Mean total HoNOSCA score was 19.40 (±8.17) on a scale from 0 to 52, indicative of a severity similar to that at inpatient admission.[21,22]

For peer review only - http://bmjopen.bmj.com/site/about/guidelines.xhtml

The proportion of the included sample notified from each region within the British Isles is reported in Table 2, alongside the population of young people in each region by age. England has the largest population (78%) and notified 70% of new cases. Scotland, containing only 7% of the total population, notified 14% of the sample and Northern Ireland, containing only 3% of the population, notified 13% of the sample. By contrast, the Republic of Ireland notified only 2% of cases, despite containing 8% of the population, and Wales notified no eligible cases (some cases were notified but did not meet inclusion criteria), despite containing 4% of the population.

Incidence rates

Table 3 details observed incidence rates (IR0) and adjusted incidence rates (IR1 and IR2) by age. Incidence rates for the total sample ranged from a minimum of 5.75 per 100,000 young people (95% CI 5.23 to 6.30; IR0) to a maximum of 18.22 per 100,000 young people (95% CI 17.29 to 19.18; IR2), with IR1, the rate hypothesised to be the most accurate, falling between these two values at 13.68 per 100,000 population (95% CI 12.88 to 14.52). Focusing on IR1 rates, total incidence increased steadily with age, peaking at 16 (30.37, 95% CI 26.70 to 34.41), with a substantial drop at the age of 17 (14.35, 95% CI 11.88 to 17.19).

Table 4 reports incidence rates by age and sex. Incidence among young men followed a similar pattern to overall incidence rates reported in Table 3, being highest at the age of 16 (5.14) and half that at age 17 (2.54). The highest incidence among young women was seen a year earlier than for boys, at the age of 15 (57.77), with similar rates at age 16 (56.95), dropping by more than half at age 17 (26.82).

Discussion

Principal findings

This study provides up-to-date estimates of incident cases of anorexia nervosa in young people aged 8 to 17 presenting to CAMHS services in the UK and Republic of Ireland. Our mid-range, missing data-adjusted estimate (IR1) of the incidence of anorexia nervosa in the full sample of young people aged 8 to 17 years was approximately 14 per 100,000.

For peer review only - http://bmjopen.bmj.com/site/about/guidelines.xhtml

Comparison with other studies

This result is lower than previous primary care-based estimates of 18–20 per 100,000 focusing on young people aged 10–19.[2,5] This difference is due to the different age ranges in the studies; the inclusion of children as young as 8 in the current study, who have relatively low incidence, and exclusion of adolescents aged 18 to 19, whose incidence is relatively high, makes the results difficult to compare. However, comparing rates for 10 to 14-year olds, available in both studies, produces similar incidence rates, with rates of 12.6 per 100,000 in the current study, compared to 13.1 per 100,000 in 2009.[2] For females, the rates are 23.3 per 100,000 in the current study compared to 24.0 per 100,000 in 2009 and for males, 2.4 and 2.5 per 100,000, respectively. However, this comparison should be treated with caution given the very different settings – primary care versus secondary care.

Existing secondary care estimates of the incidence of anorexia nervosa in the UK are limited to children under the age of 13, with an overall incidence of 1.09 per 100,000 reported for children aged between 6 and 13 between 2005 and 2006.[7] The methodology for this study was very similar to the CostED methodology, using the CAPSS system but additionally the British Paediatric Surveillance System. For comparison with the current study, the incidence rate for children between 8 and 13 was approximately 1.5 per 100,000 for DSMIV anorexia nervosa or 1.8 per 100,000 for DSMIV anorexia nervosa plus 'other eating disorders' likely to contain cases that would now be classified as anorexia nervosa using DSM5. This compares to a rate of 5.83 per 100,000 in the current study for children of the same age. This estimate is substantially higher than the 2006 estimates suggesting that incidence rates for younger children have increased over time.

Strengths and weaknesses of the study

The large, nationally representative sample of this study is a strength. It included young people with anorexia nervosa, diagnosed using DSM5 criteria, from across the UK and the Republic of Ireland and thus avoided biases inherent in studying clinical samples via a small number of centres in a limited number of geographical areas.

With only a 50% response rate from CAPSS clinicians and a third of questionnaires not returned, missing data were a constraint. There are many reasons why clinicians may fail to return

For peer review only - http://bmjopen.bmj.com/site/about/guidelines.xhtml

notification cards or questionnaires, including changes in place of employment, competing priorities, or the belief that cases will be reported by a colleague.[13] This problem was addressed by adjusting the observed incidence rates using assumptions about incidence among both missing case notifications and missing questionnaires.

The methodology is also limited to young people seen by child and adolescent psychiatrists. Cases that would not be identified by this methodology include those who have not come to the attention of services, those managed by general practitioners in primary care, and those in the care of mental health services without psychiatric input, such as nurse-led facilities. This latter concern was an issue in Northern Ireland where, due to initial low numbers of notifications, investigation by the research team identified a number of nurse-led facilities which were invited to contribute, and subsequently reported just over half of all cases in Northern Ireland. In terms of missing primary care cases, given UK guidelines for assessment and diagnosis of anorexia nervosa to be carried out by child and adolescent psychiatrists in secondary care settings,[10] it is reasonable to assume that many of those cases remaining in primary care would not meet criteria for DSM5 anorexia nervosa.

Meaning of the study

These results provide up-to-date estimates of the incidence of anorexia nervosa in young people. Whilst firm conclusions relating to changes in incidence rates over time for the entire sample cannot be drawn due to lack of existing secondary care evidence, service providers and commissioners should consider evidence to suggest an increase in incidence in younger children.

Unanswered questions and future research

Future research should explore the development of earlier interventions, given evidence of an increase in incidence in young children suggesting that onset of anorexia nervosa may be starting earlier for some young people than suggested by previous research. Research is also needed to identify approaches to the assessment of incidence simultaneously in primary and secondary care.

For peer review only - http://bmjopen.bmj.com/site/about/guidelines.xhtml

Word count

3686

Acknowledgements

The authors would like to thank all the clinicians who were kind enough to give up their time to provide the CostED study with CAPSS notifications and to complete questionnaires. We know your time is precious and in short supply and we are truly grateful. We would also like to thank the team at the Child and Adolescent Psychiatry Surveillance System, based at the Royal College of Psychiatrist's Centre for Care Quality and Improvement, for their support with the design and running of the surveillance aspect of this study. Finally, we would like to thank the members of the CostED study steering committee for their time and their support.

For peer review only - http://bmjopen.bmj.com/site/about/guidelines.xhtml

Contributors

SB was principal investigator, led the study and managed the King's College London research staff. GM managed the Northern Ireland research staff. SB and IE conceived the study. SB, IE, GM, MS, DN, TF, SG and BB designed the study. MS, DS, TF, IE, SG provided clinical expertise. DN and TF provided expertise on CAPSS methodology. HP, RS, NL and GK contributed to data collection, data entry and data cleaning. SB, HP, MP and BB carried out the data analysis. SB and HP drafted the manuscript. All authors have seen, commented on and approved the final version of the manuscript.

Funding

The study was funded by the NIHR Health Service & Delivery Research programme (11/1023/17). The study funders had no role in the study design, the collection, analysis or interpretation of data, the writing of the report, or the decision to submit the article for publication. The authors were independent from the funders and all authors had full access to all of the data in the study and can take responsibility for the integrity of the data and the accuracy of the data analysis.

Disclaimer

The views expressed are those of the authors and not necessarily those of the NHS, the NIHR or the Department of Health and Social Care.

Competing interests

TF reports she is Chair of the Child and Adolescent Psychiatry Surveillance Service that was used to run part of the study, which is an unpaid position (other than travel expenses). KJ reports that he was principal investigator for the Aberdeen site for a Sunovion sponsored multisite trial on effectiveness of Lurasidone in paediatric schizophrenia. No other authors report conflicts of interest.

Data sharing

No additional data available

For peer review only

References

1 Arcelus J, Mitchell AJ, Wales J, Nielsen S. Mortality rates in patients with anorexia nervosa and other eating disorders: A meta-analysis of 36 Studies. Arch Gen Psychiatry 2011;68:724–731.

2 Micali N, Hagberg KW, Petersen I, Treasure JL. The incidence of eating disorders in the UK in 2000-2009: findings from the General Practice Research Database. *BMJ Open* 2013;3:e002646.

3 Hoek HW. Incidence, prevalence and mortality of anorexia nervosa and other eating disorders. *Curr Opin Psychiatry* 2006;19:389–394.

4 Smink FRE, van Hoeken D, Hoek HW. Epidemiology of eating disorders: incidence, prevalence and mortality rates. Current Psychiatry Reports 2012;14:406–414.

5 Currin L, Schmidt U, Treasure J, Jick H. Time trends in eating disorder incidence. *Br J Psychiatry* 2005;186:132–135.

6 House J, Schmidt U, Craig M, et al. Comparison of specialist and nonspecialist care pathways for adolescents with anorexia nervosa and related eating disorders. *Int J Eat Disord* 2012;45:949–956.

7 Nicholls DE, Lynn R, Viner RM. Childhood eating disorders: British national surveillance study. *Br J Psychiatry* 2011;98:295–301.

8 Lask B, Bryant-Waugh R, Wright F, Campbell M, Willoughby K, Waller G. Family physician consultation patterns indicate high risk for early-onset anorexia nervosa. *Int J Eat Disord* 2005;38:269–272.

9 Goodman R, Ford T, Simmons H, Gatward R, Meltzer H. Using the Strengths and Difficulties Questionnaire (SDQ) to screen for child psychiatric disorders in a community sample. *Br J Psychiatry* 2000;177:534–539.

10 National Institute for Health and Care Excellence. Eating disorders: recognition and treatment. NICE guideline [NG69]. National Institute for Health and Care Excellence, 2017.

11 Byford S, Petkova H, Stuart R, et al. Cost-effectiveness of models of care for young people with eating disorders (the CostED study). *Health Service and Delivery Research* 2018 (in press).

For peer review only - http://bmjopen.bmj.com/site/about/guidelines.xhtml

12 Lynn RM, Viner RM, Nicholls DE. Ascertainment of early onset eating disorders: a pilot for developing a national child psychiatric surveillance system. *Child Adolesc Ment Health* 2012;17:109–112.

13 Knowles RL, Friend H, Lynn R, Mitchell S, Michie C, Ihekweazu C. Surveillance of rare diseases: a public health evaluation of the British Paediatric Surveillance Unit. *J Public Health* 2012;34:279–286.

14 American Psychiatric Association. Diagnostic and Statistical Manual of Mental Disorders (DSM–5), Fifth Edition. Washington DC: American Psychiatric Association, 2013.

15 Le Grange D, Doyle PM, Swanson SA, Ludwig K, Glunz K, Kreipe RE. Calculation of expected body weight in adolescents with eating disorders. *Pediatrics* 2012;129:e438–e446.

16 Gowers SG, Harrington RC, Whitton A, et al. Brief scale for measuring the outcomes of emotional and behavioural disorders in children: Health of the Nation Outcome Scales for Children and Adolescents (HoNOSCA). *Br J Psychiatry* 1999;174:413–416.

17 Shaffer D, Gould MS, Brasic J, et al. A children's global assessment scale (CGAS). *Arch Gen Psychiatry* 1983;40:1228–1231.

18 Office for National Statistics. Population Estimates for UK, England and Wales, Scotland and Northern Ireland. Office for National Statistics, 2015.

19 Central Statistics Office for the Republic of Ireland. PEA11: Population estimates from 1926 by Single Year of Age, Sex and Year, https://www.cso.ie/px/pxeirestat/Statire/SelectVarVal/Define.asp?maintable=PEA11&PLanguage=0.

20 Hakulinen T, Dyba T. Precision of incidence predictions based on Poisson distributed observations. *Stat Med* 1994;13:1513–1523.

21 Gowers S, Levine W, Bailey-Rogers S, Shore A, Burhouse E. Use of a routine, self-report outcome measure (HoNOSCA-SR) in two adolescent mental health services. *Br J Psychiatry* 2002;180:266–269.

22 Gowers SG, Clark A, Roberts C, et al. Clinical effectiveness of treatments for anorexia nervosa in adolescents: Randomised controlled trial. *Br J Psychiatry* 2007;191:427–435.

For peer review only - http://bmjopen.bmj.com/site/about/guidelines.xhtml

Table 1 Characteristics of incident cases

	N	Mean (SD) or %
Age	305	14.56 (1.66)
Sex		
Female	279	91.48%
Male	26	8.52%
Ethnicity		
Any White	274	91.64%
White and Asian	6	2.01%
White and Black Caribbean	2	0.67%
White and Black African	1	0.33%
Other Mixed	1	0.33%
Indian	3	1.00%
Pakistani	2	0.67%
Bangladeshi	1	0.33%
Other Asian	4	1.34%
Black Caribbean	2	0.67%
Chinese	1	0.33%
Ethnicity not known	2	0.67%
Clinical status		
BMI	304	16.50 (2.25)
% of median expected BMI	303	83.23 (10.99)
CGAS	280	44.61 (14.08)
HoNOSCA	63	19.40 (8.17)

For peer review only

For peer review only - http://bmjopen.bmj.com/site/about/guidelines.xhtml

Table 2 Cases by region of the UK and Republic of Ireland

Region	Incident cases N	Incident cases %*	Population N	Population %
England	213	69.84%	6,194,444	77.83%
Scotland	44	14.43%	561,490	7.06%
Northern Ireland	41	13.44%	231,822	2.91%
Republic of Ireland	7	2.30%	628,251	7.89%
Wales	0	0.00%	342,627	4.31%
Total	305		7,958,634	

[*] Does not sum to 100 due to rounding

For peer review only

Table 3 Annual incidence of anorexia nervosa in young people aged 8 to 17 for 2015, reported per 100,000 young people

	Observed rate IR0		Adjusted rate IR1		Adjusted rate IR2	
Age	IR	95% CI	IR	95% CI	IR	95% CI
8	0.18	0.01 to 0.76	0.43	0.10 to 1.14	0.57	0.18 to 1.35
9	0.18	0.01 to 0.77	0.44	0.11 to 1.17	0.58	0.18 to 1.38
10	0.19	0.01 to 0.80	0.45	0.11 to 1.21	0.60	0.19 to 1.43
11	1.53	0.79 to 2.67	3.65	2.43 to 5.25	4.85	3.43 to 6.65
12	4.91	3.47 to 6.76	11.69	9.39 to 14.38	15.56	12.89 to 18.63
13	8.39	6.44 to 10.73	19.95	16.89 to 23.42	26.58	23.02 to 30.54
14	11.71	9.41 to 14.39	27.85	24.25 to 31.84	37.10	32.92 to 41.66
15	12.39	10.05 to 15.10	29.47	25.80 to 33.52	39.25	35.50 to 43.88
16	12.76	10.42 to 15.47	30.37	26.70 to 34.41	40.45	36.19 to 45.07
17	6.03	4.47 to 7.96	14.35	11.88 to 17.19	19.12	16.24 to 22.35
Total	5.75	5.23 to 6.30	13.68	12.88 to 14.52	18.22	17.29 to 19.18

For peer review only

For peer review only - http://bmjopen.bmj.com/site/about/guidelines.xhtml

Table 4 Annual incidence of anorexia nervosa in young people aged 8 to 17 for 2015 by sex, reported per 100,000 young people

	Observed incidence IR0				Adjusted incidence IR1				Adjusted incidence IR2			
Age	Female	95% CI	Male	95% CI	Female	95% CI	Male	95% CI	Female	95% CI	Male	95% CI
8	0.36	0.02 to 1.55	0.00	0.00 to 0.00	0.87	0.21 to 2.34	0.00	0.00 to 0.00	1.16	0.36 to 2.76	0.00	0.00 to 0.[illegible]
9	0.00	0.00 to 0.00	0.35	0.02 to 1.52	0.00	0.00 to 0.00	0.85	0.21 to 2.28	0.00	0.00 to 0.00	1.13	0.35 to 2.[illegible]
10	0.39	0.02 to 1.65	0.00	0.00 to 0.00	0.93	0.23 to 2.48	0.00	0.00 to 0.00	1.23	0.39 to 2.93	0.00	0.00 to 0.[illegible]
11	2.35	1.07 to 4.46	0.75	0.15 to 2.18	5.59	3.47 to 8.51	1.77	0.71 to 3.63	7.44	4.96 to 10.72	2.37	1.11 to 4.[illegible]
12	8.05	5.43 to 11.50	1.92	0.80 to 3.86	19.17	14.98 to 24.16	4.56	2.69 to 7.22	25.53	20.66 to 31.21	6.09	3.89 to 9.[illegible]
13	16.36	12.48 to 21.06	0.78	0.16 to 2.28	38.93	32.81 to 45.86	1.85	0.75 to 3.79	51.83	44.72 to 59.74	2.47	1.16 to 4.[illegible]
14	22.35	17.83 to 27.67	1.53	0.56 to 3.32	53.19	46.08 to 61.10	3.64	2.00 to 6.07	70.84	62.58 to 79.88	4.84	2.91 to 7.[illegible]
15	24.28	19.59 to 29.74	1.11	0.33 to 2.71	57.77	50.41 to 65.90	2.65	1.31 to 4.78	76.93	68.39 to 86.23	3.54	1.95 to 5.[illegible]
16	23.94	19.36 to 29.28	2.16	0.99 to 4.11	56.95	49.75 to 64.90	5.14	3.20 to 7.83	75.87	67.52 to 84.97	6.85	4.57 to 9.[illegible]
17	11.27	8.22 to 15.08	1.07	0.32 to 2.60	26.82	21.98 to 32.40	2.54	1.25 to 4.58	35.71	30.09 to 42.07	3.39	1.87 to 5.[illegible]
Total	10.78	9.77 to 11.87	0.96	0.68 to 1.30	25.66	24.09 to 27.30	2.28	1.84 to 2.79	34.17	32.36 to 36.06	3.03	2.52 to 3.[illegible]

For peer review only - http://bmjopen.bmj.com/site/about/guidelines.xhtml

For peer review only

Figure 1 Flow diagram of case ascertainment

For peer review only

For peer review only - http://bmjopen.bmj.com/site/about/guidelines.xhtml

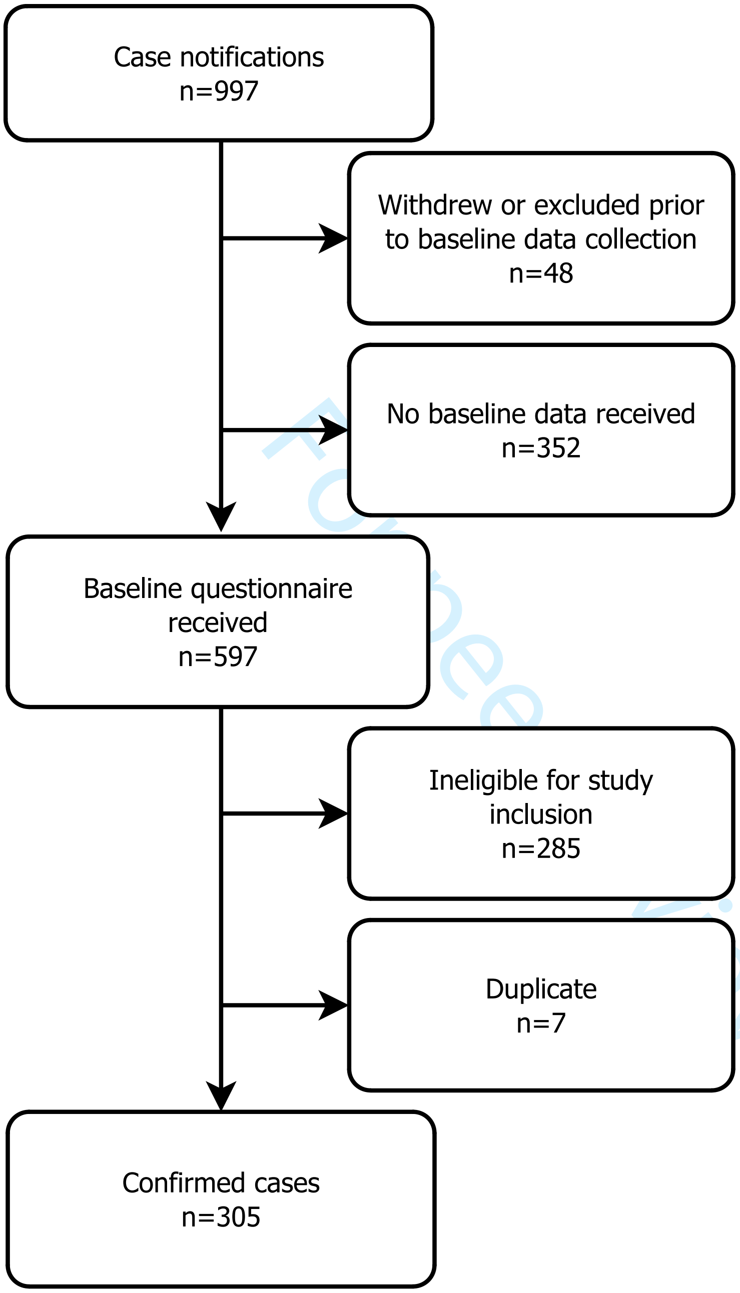

For peer review only - http://bmjopen.bmj.com/site/about/guidelines.xhtml

Web extras

Case notification definition

Please report any child/young person aged 8 to 17 years and 11 months inclusive, who meets the case notification definition criteria below for the first time in the last month. One bullet point criterion from each group below should be fulfilled.

Group A

- Restriction of food, low body weight, or
- Weight less than expected for age

Group B

- Fear of gaining weight, or
- Fear of becoming fat, or
- Behaviour that interferes with weight gain, for example excessive exercising, self-induced vomiting, use of laxatives and diuretics

Group C

- Body image disturbance, or
- Persistent lack of recognition of the seriousness of the current low body weight

Exclusions

- Patients who are not underweight
- Patients with bulimia nervosa, binge eating disorder, avoidant restrictive food intake disorder or other failure to thrive presentations

For peer review only

STROBE Statement—Checklist of items that should be included in reports of ***cross-sectional studies***

	Item No	Recommendation	Page No
**Title and abstract**	1	(*a*) Indicate the study's design with a commonly used term in the title or the abstract	1
		(*b*) Provide in the abstract an informative and balanced summary of what was done and what was found	2
**Introduction**			
Background/rationale	2	Explain the scientific background and rationale for the investigation being reported	4
Objectives	3	State specific objectives, including any prespecified hypotheses	4
**Methods**			
Study design	4	Present key elements of study design early in the paper	4-5
Setting	5	Describe the setting, locations, and relevant dates, including periods of recruitment, exposure, follow-up, and data collection	4-5
Participants	6	(*a*) Give the eligibility criteria, and the sources and methods of selection of participants	5, 7
Variables	7	Clearly define all outcomes, exposures, predictors, potential confounders, and effect modifiers. Give diagnostic criteria, if applicable	6, 7
Data sources/ measurement	8*	For each variable of interest, give sources of data and details of methods of assessment (measurement). Describe comparability of assessment methods if there is more than one group	6
Bias	9	Describe any efforts to address potential sources of bias	n/a
Study size	10	Explain how the study size was arrived at	n/a – population level
Quantitative variables	11	Explain how quantitative variables were handled in the analyses. If applicable, describe which groupings were chosen and why	8-10
Statistical methods	12	(*a*) Describe all statistical methods, including those used to control for confounding	10
		(*b*) Describe any methods used to examine subgroups and interactions	n/a
		(*c*) Explain how missing data were addressed	8-9
		(*d*) If applicable, describe analytical methods taking account of sampling strategy	n/a
		(*e*) Describe any sensitivity analyses	n/a
**Results**			
Participants	13*	(a) Report numbers of individuals at each stage of study—eg numbers potentially eligible, examined for eligibility, confirmed eligible, included in the study, completing follow-up, and analysed	10, 23
		(b) Give reasons for non-participation at each stage	10, 23
		(c) Consider use of a flow diagram	23
Descriptive data	14*	(a) Give characteristics of study participants (eg demographic, clinical, social) and information on exposures and potential confounders	10-11, 19
		(b) Indicate number of participants with missing data for each	19

For peer review only

		variable of interest	
Outcome data	15*	Report numbers of outcome events or summary measures	n/a
Main results	16	(*a*) Give unadjusted estimates and, if applicable, confounder-adjusted estimates and their precision (eg, 95% confidence interval). Make clear which confounders were adjusted for and why they were included	11, 21-22
		(*b*) Report category boundaries when continuous variables were categorized	n/a
		(*c*) If relevant, consider translating estimates of relative risk into absolute risk for a meaningful time period	n/a
Other analyses	17	Report other analyses done—eg analyses of subgroups and interactions, and sensitivity analyses	n/a
**Discussion**			
Key results	18	Summarise key results with reference to study objectives	12
Limitations	19	Discuss limitations of the study, taking into account sources of potential bias or imprecision. Discuss both direction and magnitude of any potential bias	12-13
Interpretation	20	Give a cautious overall interpretation of results considering objectives, limitations, multiplicity of analyses, results from similar studies, and other relevant evidence	13, 12
Generalisability	21	Discuss the generalisability (external validity) of the study results	13
**Other information**			
Funding	22	Give the source of funding and the role of the funders for the present study and, if applicable, for the original study on which the present article is based	15

*Give information separately for exposed and unexposed groups.

Note: An Explanation and Elaboration article discusses each checklist item and gives methodological background and published examples of transparent reporting. The STROBE checklist is best used in conjunction with this article (freely available on the Web sites of PLoS Medicine at http://www.plosmedicine.org/, Annals of Internal Medicine at http://www.annals.org/, and Epidemiology at http://www.epidem.com/). Information on the STROBE Initiative is available at www.strobe-statement.org.

For peer review only

BMJ Open

Incidence of anorexia nervosa in young people in the United Kingdom and the Republic of Ireland: A national surveillance study

Journal:	*BMJ Open*
Manuscript ID	bmjopen-2018-027339.R1
Article Type:	Research
Date Submitted by the Author:	25-Mar-2019
Complete List of Authors:	Petkova, Hristina; Institute of Psychiatry Psychology and Neuroscience, Health Service and Population Research Department<br>Simic, Mima; South London and Maudsley NHS Foundation Trust<br>Nicholls, Dasha; Imperial College London<br>Ford, Tamsin; University of Exeter<br>Prina, A.Matthew; Institute of Psychiatry Psychology and Neuroscience<br>Stuart, Ruth; Institute of Psychiatry Psychology and Neuroscience, Health Service and Population Research Department<br>Livingstone, Nuala; Queen's University Belfast<br>Kelly, Grace; Queen's University Belfast<br>Macdonald, Geraldine; University of Bristol<br>Eisler, Ivan; South London and Maudsley NHS Foundation Trust<br>Gowers, Simon; University of Liverpool<br>Barrett, Barbara; Institute of Psychiatry Psychology and Neuroscience<br>Byford, Sarah; Institute of Psychiatry Psychology and Neuroscience
**Primary Subject Heading**:	Mental health
Secondary Subject Heading:	Epidemiology
Keywords:	Eating disorders < PSYCHIATRY, Child & adolescent psychiatry < PSYCHIATRY, EPIDEMIOLOGY, anorexia nervosa

SCHOLARONE™
Manuscripts

Incidence of anorexia nervosa in young people in the United Kingdom and the Republic of Ireland: A national surveillance study

Hristina Petkova, Institute of Psychiatry, Psychology & Neuroscience at King's College London (Research Fellow).

Mima Simic, South London and Maudsley NHS Foundation Trust (Consultant Child and Adolescent Psychiatrist).

Dasha Nicholls, Imperial College London (Reader in Child and Adolescent Psychiatry).

Tamsin Ford, University of Exeter Medical School (Professor of Child and Adolescent Psychiatry).

A. Matthew Prina, Institute of Psychiatry, Psychology & Neuroscience at King's College London (Senior Lecturer in Epidemiology).

Ruth Stuart, Institute of Psychiatry, Psychology & Neuroscience at King's College London (Research Assistant).

Nuala Livingstone, Queen's University Belfast (Research Fellow).

Grace Kelly, Queen's University Belfast (Research Fellow).

Geraldine Macdonald, University of Bristol (Professor of Social Work).

Ivan Eisler, South London and Maudsley NHS Foundation Trust (Professor of Family Psychology and Family Therapy).

Simon Gowers, University of Liverpool (Professor of Adolescent Psychiatry).

Barbara Barrett, Institute of Psychiatry, Psychology & Neuroscience at King's College London (Senior Lecturer in Health Economics).

Sarah Byford, Institute of Psychiatry, Psychology & Neuroscience at King's College London (Professor of Health Economics).

Corresponding author: Sarah Byford, Institute of Psychiatry, Psychology & Neuroscience at King's College London, P024 David Goldberg Centre, De Crespigny Park, London SE5 8AF, UK, s.byford@kcl.ac.uk, 02078480043

For peer review only

Abstract

Objectives: This study aimed to estimate the incidence of DSM5 anorexia nervosa in young people in contact with child and adolescent mental health services in the UK and Republic of Ireland.

Design: Observational, surveillance study using the Child and Adolescent Psychiatry Surveillance System, involving monthly reporting by child and adolescent psychiatrists between 1st February 2015 and 30th September 2015.

Setting: UK and Republic of Ireland

Participants: Clinician-reported data on young people aged 8 to 17 in contact with child and adolescent mental health services for a first episode of anorexia nervosa.

Main outcome measures: Annual incidence rates estimated as confirmed new cases per 100,000 population at risk.

Results: 305 incident cases of anorexia nervosa were reported over the 8-month surveillance period and assessed as eligible for inclusion. The majority were young women (91%), from England (70%), and of white ethnicity (92%). Mean age was 14.6 years (±1.66) and mean percentage of median expected BMI for age and sex was 83.23% (±10.99%). The overall incidence rate, adjusted for missing data, was estimated to be 13.68 per 100,000 population (95% CI 12.88 to 14.52), with rates of 25.66 for young women (95% CI 24.09 to 27.30) and 2.28 for young men (95% CI 1.84 to 2.79). Incidence increased steadily with age, peaking at 15 for young women (57.77, 95% CI 50.41 to 65.90) and 16 for young men (5.14, 95% CI 3.20 to 7.83). Comparison with earlier estimates suggests incidence rates for children aged 13 and under have increased over the last ten years.

Conclusions: These results provide up-to-date estimates of the incidence of anorexia nervosa in young people. Service providers and commissioners should consider evidence to suggest an increase in incidence in younger children.

Study registration: International Standard Randomised Controlled Trial Number Register [ISRCTN12676087].

Strengths and limitations of this study

- This study provides up-to-date estimates of the incidence of anorexia nervosa in young people aged 8 to 17 presenting to child and adolescent mental health services in the UK and Republic of Ireland.

For peer review only - http://bmjopen.bmj.com/site/about/guidelines.xhtml

- The study benefits from a large, nationally representative sample from across the UK and the Republic of Ireland.
- Results were limited by missing data which was dealt with by adjusting observed incidence rates using assumptions about incidence among missing cases.
- Results are relevant to young people diagnosed with anorexia nervosa by child and adolescent psychiatrists and not to those who are managed by general practitioners in primary care or those who have not come to the attention of services, for example those who choose not to seek help.

For peer review only

For peer review only - http://bmjopen.bmj.com/site/about/guidelines.xhtml

What is already known on this topic

Estimates of the incidence of anorexia nervosa in the UK and Republic of Ireland are at least ten years old. In addition, most estimates are derived from community-based primary care records, which fail to accurately record all new cases, since cases may present to accident and emergency and require immediate paediatric or psychiatric input, including inpatient admission. Existing incidence data from secondary care settings in the UK are limited and no data were identified for the Republic of Ireland. The only incidence study identified which used secondary care data – a British national surveillance study – focused only on children under 13 with data collected over ten years ago (2005-2006).

What this study adds

This study provides estimates of the incidence of DSM5 anorexia nervosa in young people aged 8 to 17 years in contact with child and adolescent mental health services in the United Kingdom and Republic of Ireland, which replace estimates that are over ten years old and limited by age range or geographical coverage. The results suggest that overall incidence rates have remained steady, but rates for young people under the age of 13 have increased over time. Providing up-to-date incidence estimates and monitoring trends can help to support health service planning for the provision of timely, effective and cost-effective interventions.

For peer review only - http://bmjopen.bmj.com/site/about/guidelines.xhtml

For peer review only

Introduction

Anorexia nervosa is a serious and enduring eating disorder with high morbidity and the highest mortality among psychiatric disorders.[1] Young women are particularly susceptible, with annual UK estimates of 37 new diagnoses of anorexia nervosa per 100,000 for girls aged 10 to 19 years, compared to 3 per 100,000 for boys of the same age.[2] Prevalence estimates in young people range from 0.3% to 0.6%.[3–4]

Accurate epidemiological estimates of the number of new anorexia nervosa cases per year and their sex and age profile are needed for causal investigations and service planning.[2] However, available estimates in the UK are at least ten years old.[2,5–7] In addition, most estimates are derived from community-based primary care records,[2,5] which fail to accurately record all new cases.[8,9] Undetected anorexia nervosa cases may present to accident and emergency and require immediate paediatric or psychiatric input, including inpatient admission. Some young people may therefore bypass primary care and, consistent with UK guidelines,[10] are likely to be assessed and diagnosed by a child and adolescent psychiatrist in a secondary care setting, making secondary care records a more reliable source of data on anorexia nervosa incidence than primary care registers.

Existing incidence data from secondary care settings in the UK are limited. One study, focusing only on adolescents aged 13 to 18, was limited to Greater London and reported presentation rates to child and adolescent mental health services (CAMHS), rather than incidence estimates.[6] A second study, which used a national surveillance design, focused only on children under 13.[7] The current study aimed to estimate the incidence of anorexia nervosa in secondary care services for young people between the ages of 8 and 17 years in the UK and the Republic of Ireland. This work formed part of a study exploring the cost-effectiveness of models of care for young people with eating disorders (the CostED study).[11]

Methods

Design

An observational, surveillance study was undertaken using the Child and Adolescent Psychiatry Surveillance System (CAPSS). CAPSS is a system designed to ascertain cases of rare childhood mental health conditions in the UK and Republic of Ireland through monthly reporting by clinicians and relies on non-consent to maximise the accuracy of

For peer review only - http://bmjopen.bmj.com/site/about/guidelines.xhtml

epidemiological estimates. The CAPSS system has been operating since 2009[12] and is based on the well-established British Paediatric Surveillance Unit (BPSU) system.[13]

The study was approved by the CAPSS Executive Committee, King's College London Research Ethics Committee [PNM/13/14-105], and the Health Research Authority Confidentiality Advisory Group [CAG 4-03(PR1)/2014] under Section 251 of the NHS Act 2006, which enables disclosure of confidential patient information for purposes where it is not possible to use anonymised information and where seeking consent is not practical.

Inclusion and exclusion criteria

The study included young people between 8 and 17 years of age, in contact with CAMHS for a first episode of anorexia nervosa according to DSM5 diagnostic criteria.[14] Anorexia nervosa is exceptionally rare in children under 8 and the cut-off at 17 was due to the focus on young people in contact with CAMHS, with many young people transitioning to adult services at the age of 18. New cases were notified for a period of eight months from 1st February to 30th September 2015. Cases whose clinician-reported data were insufficient to assess eligibility were excluded, as were duplicate cases notified more than once by the same or different clinicians.

Procedures

At the time of the study, CAPSS used a report card, known as the yellow card, containing a list of conditions being surveyed. Yellow cards, along with reporting instructions and protocols for new studies, are sent monthly from the CAPSS office to a mailing list of all hospital, university and community child and adolescent consultant psychiatrists across the United Kingdom and the Republic of Ireland. Reporting clinicians are asked to check boxes against any of the reportable conditions they have seen in the preceding month, or to check a "nil return" box and return the card to CAPSS. A tear-off slip is provided for respondents to keep a record of the patients reported. "Positive" returns are allocated a unique CAPSS ID number and notified to the appropriate research investigator, who then contacts the reporting clinician directly to request completion of a questionnaire using the CAPSS ID to enable the clinician to identify the relevant patient.

For the CostED study, the yellow card contained a check box for anorexia nervosa and was sent to clinicians along with a protocol card detailing the case notification definition for

For peer review only - http://bmjopen.bmj.com/site/about/guidelines.xhtml

anorexia nervosa. The case notification definition (see web extras) was based on DSM5 diagnostic criteria for anorexia nervosa and was intended to aid clinicians in their decision to tick "yes" or "no" on the yellow card. It was not intended to identify whether a case met study inclusion criteria, which was determined by the research group after receipt of all necessary data.

Data

Questionnaires were sent to clinicians who reported a positive case of anorexia nervosa, identified via the unique CAPSS ID number. Questionnaires were completed from clinical records and clinicians were asked to provide data relating to the time the case was initially assessed and diagnosed. The questionnaire covered clinical features to enable assessment of case eligibility, referral pathway information to ensure assessment and diagnosis had not happened prior to the study surveillance period, and a limited set of standard patient identifiers in line with CAPSS procedures and ethics requirements, which were used to describe the sample and to identify duplicate notifications. In addition, clinicians were asked to confirm whether the case was a first episode of anorexia nervosa that had come to the attention of services.

The patient identifiers included NHS or Community Health Index (CHI) number (unique patient identifiers used in the regions of interest), hospital number, first half of postcode or town of residence for the Republic of Ireland, sex, date of birth and ethnicity (White, Mixed, Asian, Black, Chinese, Other or Unknown). In Northern Ireland, identifiers were limited to age in years and months and hospital identifier rather than hospital number, to reduce the risk of patient identification given the small geographic area. All patient identifiable data from Northern Ireland were retained by the local research team, de-duplicated, anonymised and subsequently sent to the central research team in King's College London for analysis as per requirements set out by the Northern Ireland Privacy Advisory Committee. All data storage was compliant with the EU General Data Protection Regulations.

Clinical features included: weight and height to calculate body mass index (BMI) and percentage of median expected BMI for age and sex interpreted around the 85% threshold;[15] the Health of the Nation Outcome Scales for Children and Adolescents (HoNOSCA[16]), a routine outcome measure rating 13 clinical features on a five-point severity scale including behaviours, impairments, symptoms, and social functioning of children and adolescents with

For peer review only - http://bmjopen.bmj.com/site/about/guidelines.xhtml

mental health problems; the clinician completed Children's Global Assessment Scale (CGAS[17]) used to rate emotional and behavioural functioning of young people; and a range of symptoms relating to the diagnosis of anorexia nervosa.

Unreturned or incomplete questionnaires were chased via email and telephone. Cases where any symptom required for case definition was absent despite chasing, were assessed for eligibility by a consultant child and adolescent psychiatrist (MS).

Case eligibility

Cases were assessed as eligible for the study if: (a) they were between 8 and 17 years of age; (b) they had no previous episode of anorexia nervosa that had come to the attention of services; (c) they received a clinical assessment in the reporting service during the study surveillance period; (d) they had not been referred from another secondary health service (to ensure assessment and diagnosis had not happened prior to study surveillance period); and (e) the following clinical symptoms were present: "Restriction of energy intake relative to requirements" and "Persistent behaviour that interferes with weight gain, despite low weight". This broad definition was subsequently checked using a tighter DSM5 analytic definition including the following symptoms:

1) "Restriction of energy intake relative to requirements" and
2) "Intense fear of gaining weight or of becoming fat" or "Persistent behaviour that interferes with weight gain, despite low weight" and
3) "Perception that body shape/size is larger than it is" or "Preoccupation with body weight and shape" or "Lack of recognition of the seriousness of the current low body weight"

Only one case which met the broad criteria failed to meet the tighter criteria, thus confirming the validity of the broad criteria.

Removal of duplicates

Duplicates were identified by comparing NHS/CHI numbers, hospital numbers/ hospital identifiers and date of birth/age in years and months, as appropriate. The management of duplicates depended upon the outcome for the original notification for which a duplicate was identified. Four scenarios were considered: (1) duplicates where the original notification met study inclusion criteria were excluded and the original retained; (2) duplicates where the original notification had been excluded because the young person was under 8 years of age or

For peer review only - http://bmjopen.bmj.com/site/about/guidelines.xhtml

did not meet the clinical criteria were assessed as a new case to determine if the case now met eligibility criteria; (3) duplicates where the original notification was excluded due to a previous episode of anorexia nervosa, a diagnosis date prior to the study surveillance period or referral from another secondary care service, were excluded; and (4) duplicates where the original notification contained insufficient information to judge eligibility were checked to see if the duplicate contained the missing information and, if available, the original notification was reassessed for eligibility and the duplicate managed as per the scenarios above.

Data analysis

Data analysis was performed using Stata IC v14.2 and Microsoft Excel 2010. Observed incidence rates (denoted IR0), defined as the number of new cases during a specified period of time in a population at risk for developing the disease, were calculated as follows: the number of confirmed new cases of anorexia nervosa in the 8-month surveillance period converted to 12 months [(N cases over 8 months/8)*12], divided by the population at risk and multiplied by 100,000 to give the rate per 100,000 young people.

*IR0 = (confirmed new cases converted to 12 months)/the population at risk *100,000*

The population at risk was calculated as the total number of children of each year of age and each sex in the UK and Republic of Ireland minus the number of prevalent cases who, once diagnosed, are no longer part of the "at risk" population. Population data for 2015 were obtained from the Office for National Statistics for the UK[18] and the Central Statistics Office for the Republic of Ireland.[19] To estimate the number of prevalent cases each year, incident cases in the previous age band were used as a proxy. For example, incident cases aged eight were used as a proxy for prevalent cases in the estimation of the 'at risk' population aged nine, and so on.

To consider incidence among unobserved missing cases, adjustments were needed for unreturned CAPSS notification cards and questionnaires. For CAPSS notification cards, just over half of all notification cards sent out were returned (50.16%). To account for incidence among the 49.84% of unreturned cards, two assumptions were made, and an appropriate correction applied to IR0, the observed incidence rate:

For peer review only - http://bmjopen.bmj.com/site/about/guidelines.xhtml

Assumption 1: To take into consideration the possibility that unreturned cards are more likely to be 'nil' returns, it was assumed that half of unreturned cards (24.92%) were 'negative' and half followed the same proportion of 'negative' and 'positive' as the returned cards. This assumption translates into a correction coefficient of 1.50 derived from (24.92+50.16)/50.16.

Assumption 2: Making no assumptions of bias in the likelihood of unreturned cards being either positive or negative returns, it was assumed that all unreturned cards followed the same proportion of 'negative' and 'positive' as returned cards. This assumption translates into a correction coefficient of 1.99 derived from (49.84+50.16)/50.16.

These assumptions provide a range of incidence rates, from a minimum (observed incidence rate) to a maximum (assumption 2), within which the actual rate is likely to fall. We hypothesised that assumption 1 provides the most realistic estimate since it assumes a bias in the response rates with greater likelihood that unreturned cards are negative ('nil' returns) but does not assume *all* unreturned cards are 'nil' returns, which is the implicit assumption within IR0.

For unreturned questionnaires, approximately two-thirds of the questionnaires that were sent to clinicians reporting positive cases of anorexia nervosa were returned (63%), leaving one-third (37%) unreturned. Since all these questionnaires relate to a 'positive' notification, we applied a correction coefficient of 1.59 derived from (37+63)/63, which assumes that the incidence rate for the unreturned questionnaires is the same as the incidence rate identified in the returned questionnaires for each year of age.

We then combined the correction coefficients described above, to generate two adjusted incidence rates:

Adjusted incidence rate 1 (IR1) = Confirmed new cases of anorexia nervosa converted to 12 months, multiplied by the correction for unreturned CAPSS notification cards under assumption 1, multiplied by the correction for unreturned questionnaires, then divided by the population at risk and multiplied by 100,000**.**

*IR1 = (confirmed new cases converted to 12 months * 1.50 * 1.59)/the population at risk *100,000*

For peer review only - http://bmjopen.bmj.com/site/about/guidelines.xhtml

Adjusted Incidence rate 2 (IR2) = Confirmed new cases of anorexia nervosa converted to 12-months, multiplied by the correction for unreturned CAPSS notification cards under assumption 2, multiplied by the correction for unreturned questionnaires and then divided by the population at risk and multiplied by 100,000.

*IR2 = (confirmed new cases converted to 12 months * 1.99 * 1.59)/the population at risk * 100,000*

For each incidence rate, IR0, IR1 and IR2, total, age-specific and sex-specific annual incidence rates for anorexia nervosa for the year 2015 and 95% confidence intervals were calculated based on the Poisson distribution[20] using the Stata command *ci means [N new anorexia nervosa cases 12m], Poisson [exposure(total population)]* for positive integers/whole incidence numbers (Stata interprets any non-integer decimal point number between 0 and 1 as the fraction of events and converts it to an integer number). Annual incidence rates were stratified by discrete age and sex.

Public and patient involvement statement

The CostED study included a patient and a parent representative on the study steering committee who contributed to the design, conduct and management of the study, including the incidence component.

Results

Case ascertainment

Case ascertainment is outlined in Figure 1. Over the eight-month surveillance period, 6401 yellow cards were sent to reporting clinicians and 3211 were returned (50%). Of these, 997 positive cases of anorexia nervosa were reported and 2214 were nil returns. Of the positive cases, 48 (5%) were excluded due to clinicans stating that they did not wish to be included in the study (due to retirement, shortage of reporting capacity etc.) or due to reporting errors. Questionnaires were sent to the remaining 949, and a further 352 positive cases (37%) were excluded as they failed to return the questionnaires, so no data were available to assess eligibility. Questionnaires were completed and returned for 597 notified cases, of which 292 (49%) were ineligible for reasons related to age, previous episode of anorexia nervosa, date of assessment outside the study's surveillance period, referral from another secondary care

For peer review only - http://bmjopen.bmj.com/site/about/guidelines.xhtml

service, insufficient information to assess diagnosis or duplicate notifications, leaving 305 incident cases of anorexia nervosa as the sample for analysis.

Demographics and clinical features

Of the 305 young people identified as having DSM5 anorexia nervosa, the majority were girls (91%), from England (70%) and of white ethnicity (92%) (see Table 1). The mean age was 14.6 years (±1.66). Clinical variables suggest these young people were significantly impaired. Mean BMI was 16.50 kg/m^2 (±2.25), where values of 16.00 to 16.99 suggesting moderate severity of anorexia nervosa. Mean percentage of median expected BMI for age and sex (the deviation from expected body weight) was 83.23% (±10.99%), falling within the range required for a diagnosis of anorexia nervosa (<85%). Mean CGAS score was 44.61 (±14.08), which falls within the range for 'obvious problems' (41–50) on a scale from 1 to 100 (1 being the worst and 100 the best emotional and behavioural functioning). Mean total HoNOSCA score was 19.40 (±8.17) on a scale from 0 to 52, indicative of a severity similar to that at inpatient admission.[21,22]

The proportion of the included sample notified from each region within the British Isles is reported in Table 2, alongside the population of young people in each region by age. England has the largest population (78%) and notified 70% of new cases. Scotland, containing only 7% of the total population, notified 14% of the sample and Northern Ireland, containing only 3% of the population, notified 13% of the sample. By contrast, the Republic of Ireland notified only 2% of cases, despite containing 8% of the population, and Wales notified no eligible cases (some cases were notified but did not meet inclusion criteria), despite containing 4% of the population.

Incidence rates

Table 3 details observed incidence rates (IR0) and adjusted incidence rates (IR1 and IR2) by age. Incidence rates for the total sample ranged from a minimum of 5.75 per 100,000 young people (95% CI 5.23 to 6.30; IR0) to a maximum of 18.22 per 100,000 young people (95% CI 17.29 to 19.18; IR2), with IR1, the rate hypothesised to be the most accurate, falling between these two values at 13.68 per 100,000 population (95% CI 12.88 to 14.52). Focusing on IR1 rates, total incidence increased steadily with age, peaking at 16 (30.37, 95% CI 26.70 to 34.41), with a substantial drop at the age of 17 (14.35, 95% CI 11.88 to 17.19).

For peer review only - http://bmjopen.bmj.com/site/about/guidelines.xhtml

Table 4 reports incidence rates by age and sex. Incidence among young men followed a similar pattern to overall incidence rates reported in Table 3, being highest at the age of 16 (5.14) and half that at age 17 (2.54). The highest incidence among young women was seen a year earlier than for boys, at the age of 15 (57.77), with similar rates at age 16 (56.95), dropping by more than half at age 17 (26.82).

Discussion

Principal findings

This study provides up-to-date estimates of incident cases of anorexia nervosa in young people aged 8 to 17 presenting to CAMHS services in the UK and Republic of Ireland. Our mid-range, missing data-adjusted estimate (IR1) of the incidence of anorexia nervosa in the full sample of young people aged 8 to 17 years was approximately 14 per 100,000.

Comparison with other studies

This result is lower than previous primary care-based estimates of 18–20 per 100,000 focusing on young people aged 10–19.[2,5] This difference is due to the different age ranges in the studies; the inclusion of children as young as 8 in the current study, who have relatively low incidence, and exclusion of adolescents aged 18 to 19, whose incidence is relatively high, makes the results difficult to compare. However, comparing rates for 10 to 14-year olds, available in both studies, produces similar incidence rates, with rates of 12.6 per 100,000 in the current study, compared to 13.1 per 100,000 in 2009.[2] For females, the rates are 23.3 per 100,000 in the current study compared to 24.0 per 100,000 in 2009 and for males, 2.4 and 2.5 per 100,000, respectively. However, this comparison should be treated with caution given the very different settings – primary care versus secondary care.

Existing secondary care estimates of the incidence of anorexia nervosa in the UK are limited to children under the age of 13, with an overall incidence of 1.09 per 100,000 reported for children aged between 6 and 13 between 2005 and 2006.[7] The methodology for this study was very similar to the CostED methodology, using the CAPSS system but additionally the British Paediatric Surveillance System. For comparison with the current study, the incidence rate for children between 8 and 13 was approximately 1.5 per 100,000 for DSMIV anorexia nervosa or 1.8 per 100,000 for DSMIV anorexia nervosa plus 'other eating disorders' likely to contain cases that would now be classified as anorexia nervosa using DSM5. This compares to a rate of 5.83 per 100,000 in the current study for children of the same age. This estimate is

For peer review only - http://bmjopen.bmj.com/site/about/guidelines.xhtml

substantially higher than the 2006 estimates suggesting that incidence rates for younger children have increased over time.

The results presented are also supported by international evidence. One study carried out in Italy demonstrated a significant reduction in age at onset for anorexia nervosa in consecutive outpatient referrals between 1985 and 2008 (n=1,666).[23] A second study exploring time trends in the incidence of anorexia nervosa, which was carried out using data from the Norwegian National Patient Register, found overall rates of anorexia nervosa to be stable between 2010 and 2016 for the sample as a whole, but increasing for young females aged between 10 and 14.[24]

Strengths and weaknesses of the study

The large, nationally representative sample of this study is a strength. The study included young people with anorexia nervosa, diagnosed using DSM5 criteria, from across the UK and the Republic of Ireland and thus avoided biases inherent in studying clinical samples via a small number of centres in a limited number of geographical areas. The results are of relevance primarily to the UK and Republic of Ireland but may be of value to other high-income countries.

With only a 50% response rate from CAPSS clinicians and a third of questionnaires not returned, missing data were a major constraint. There are many reasons why clinicians may fail to return notification cards or questionnaires, including changes in place of employment, competing priorities, or the belief that cases will be reported by a colleague.[13] This problem was addressed by adjusting the observed incidence rates using assumptions about incidence among both missing case notifications and missing questionnaires.

The methodology is also limited to young people seen by child and adolescent psychiatrists. Cases that would not be identified by this methodology include those who have not come to the attention of services, for example those who choose not to seek help, those managed by general practitioners in primary care, and those in the care of mental health services without psychiatric input, such as nurse-led facilities. This latter concern was an issue in Northern Ireland where, due to initial low numbers of notifications, investigation by the research team identified a number of nurse-led facilities which were invited to contribute, and subsequently reported just over half of all cases in Northern Ireland. In terms of missing primary care

For peer review only - http://bmjopen.bmj.com/site/about/guidelines.xhtml

cases, given UK guidelines for assessment and diagnosis of anorexia nervosa to be carried out by child and adolescent psychiatrists in secondary care settings,[10] it is reasonable to assume that many of those cases remaining in primary care would not meet criteria for DSM5 anorexia nervosa. It is also possible that current inpatient cases are under-represented; although notifications were sent to all child and adolescent psychiatrists, including those working in inpatient settings, the main focus of the CostED study was the evaluation of community-based services, and so clinicians may have mistakenly focused on notification of community-based cases.

It must also be borne in mind that service-level (rather than population-level) incidence rates are sensitive to external factors, including service availability, funding and commissioning decisions, parental and school awareness, stigma, etc., all of which will impact upon observed trends in incidence rates over time. The nature of community-based eating disorders services for children and adolescents in England has started to change following the publication of commissioning standards in June 2015,[25] as well as investment of £30 million to support the development of these services. This is unlikely to have had an impact on the CostED study data because the first allocation of funding to services was made in 2016, after the end of the CostED study surveillance period in 2015. However, these initiatives are likely to result in increases in observed incidence rates in the future.

Meaning of the study

These results provide up-to-date estimates of the incidence of anorexia nervosa in young people. Whilst firm conclusions relating to changes in incidence rates over time for the entire sample cannot be drawn due to lack of existing secondary care evidence, service providers and commissioners should consider evidence to suggest an increase in incidence in younger children.

Unanswered questions and future research

Future research should explore the development of earlier interventions, given evidence of an increase in incidence in young children suggesting that onset of anorexia nervosa may be starting earlier for some young people than suggested by previous research. Research is also needed to identify approaches to the assessment of incidence simultaneously in primary and secondary care. Multinational studies should be considered for better assessment and exploration of incidence rates in young men.

For peer review only - http://bmjopen.bmj.com/site/about/guidelines.xhtml

Word count

4089

Acknowledgements

We would like to thank all the clinicians who were kind enough to give up their time to provide the CostED study with CAPSS notifications and to complete questionnaires. We know your time is precious and in short supply and we are truly grateful. We would also like to thank the team at the Child and Adolescent Psychiatry Surveillance System, based at the Royal College of Psychiatrist's Centre for Care Quality and Improvement, for their support with the design and running of the surveillance aspect of this study, and the study steering committee for their invaluable advice.

Contributor and guarantor information

Hristina Petkova contributed to data collection, data entry, data cleaning, data analysis, and drafting of the manuscript.

Mima Simic was a co-applicant, contributed to the design of the study, provided clinical expertise, and commented on and approved the manuscript.

Dasha Nicholls was a co-applicant, contributed to the design of the study, provided expertise on CAPSS methodology, clinical expertise, and commented on and approved the manuscript.

Tamsin Ford was a co-applicant, contributed to the design of the study, provided clinical expertise, and commented on and approved the manuscript.

Matthew Prina contributed to the data analysis and commented on and approved the manuscript.

Ruth Stuart contributed to data collection, data entry, data cleaning and data analysis, and commented on and approved the manuscript.

Nuala Livingstone contributed to data collection, data entry and data management in Northern Ireland, and commented on and approved the manuscript.

Grace Kelly contributed to data collection, data entry and data management in Northern Ireland, and commented on and approved the manuscript.

Geraldine Macdonald contributed to the design of the study, managed the Northern Ireland research staff, and commented on and approved the final report.

Ivan Eisler was a co-applicant, contributed to the design of the study, provided clinical support to the research team, and commented on and approved the manuscript.

For peer review only - http://bmjopen.bmj.com/site/about/guidelines.xhtml

Simon Gowers was a co-applicant, contributed to the design of the study, provided clinical expertise, and commented on and approved the manuscript.

Barbara Barrett was a co-applicant, contributed to the design of the study, the data analysis, and commented on and approved the manuscript.

Sarah Byford was principal investigator, led the study, managed the King's College London research staff, contributed to the design of the study and the data analysis, drafted the paper, and is responsible for the overall content as guarantor.

Competing interests

Tamsin Ford reports she is Chair of the Child and Adolescent Psychiatry Surveillance Service that was used to run part of the study, which is an unpaid position (other than travel expenses). Kandarp Joshi reports that he was principal investigator for the Aberdeen site for a Sunovion sponsored multisite trial on effectiveness of Lurasidone in paediatric schizophrenia. No other authors report conflicts of interest.

Transparency statement

The lead author affirms that this manuscript is an honest, accurate, and transparent account of the study being reported; that no important aspects of the study have been omitted; and that any discrepancies from the study as planned and registered have been explained.

Role of the funding source

The study was funded by the NIHR Health Service & Delivery Research programme (11/1023/17). The study funders had no role in the study design, the collection, analysis or interpretation of data, the writing of the report, or the decision to submit the article for publication. The authors were independent from the funders and all authors had full access to all of the data in the study and can take responsibility for the integrity of the data and the accuracy of the data analysis.

Disclaimer

The views expressed are those of the authors and not necessarily those of the NHS, the NIHR or the Department of Health and Social Care.

For peer review only - http://bmjopen.bmj.com/site/about/guidelines.xhtml

Data sharing

As a result of the collection of confidential patient data without consent, and approval from the Health Research Authority (following advice from the Confidentiality Advisory Group) for data to be provided for the purposes of the specified activity only, the data cannot be made publicly available for other purposes. However, the CostED research group will consider requests for further analysis on a case by case basis, subject to appropriate ethical/HRA CAG approvals. All data enquiries should be submitted to the corresponding author for consideration in the first instance.

For peer review only

References

1 Arcelus J, Mitchell AJ, Wales J, Nielsen S. Mortality rates in patients with anorexia nervosa and other eating disorders: A meta-analysis of 36 Studies. *Arch Gen Psychiatry* 2011;68:724–731.

2 Micali N, Hagberg KW, Petersen I, Treasure JL. The incidence of eating disorders in the UK in 2000-2009: findings from the General Practice Research Database. *BMJ Open* 2013;3:e002646.

3 Hoek HW. Incidence, prevalence and mortality of anorexia nervosa and other eating disorders. *Curr Opin Psychiatry* 2006;19:389–394.

4 Smink FRE, van Hoeken D, Hoek HW. Epidemiology of eating disorders: incidence, prevalence and mortality rates. *Current Psychiatry Reports* 2012;14:406–414.

5 Currin L, Schmidt U, Treasure J, Jick H. Time trends in eating disorder incidence. *Br J Psychiatry* 2005;186:132–135.

6 House J, Schmidt U, Craig M, et al. Comparison of specialist and nonspecialist care pathways for adolescents with anorexia nervosa and related eating disorders. *Int J Eat Disord* 2012;45:949–956.

7 Nicholls DE, Lynn R, Viner RM. Childhood eating disorders: British national surveillance study. *Br J Psychiatry* 2011;98:295–301.

8 Lask B, Bryant-Waugh R, Wright F, Campbell M, Willoughby K, Waller G. Family physician consultation patterns indicate high risk for early-onset anorexia nervosa. *Int J Eat Disord* 2005;38:269–272.

9 Goodman R, Ford T, Simmons H, Gatward R, Meltzer H. Using the Strengths and Difficulties Questionnaire (SDQ) to screen for child psychiatric disorders in a community sample. *Br J Psychiatry* 2000;177:534–539.

10 National Institute for Health and Care Excellence. Eating disorders: recognition and treatment. NICE guideline [NG69]. National Institute for Health and Care Excellence, 2017.

11 Byford S, Petkova H, Stuart R, et al. Alternative community-based models of care for young people with anorexia nervosa: the CostED national surveillance study. *Health Service and Delivery Research* 2019 (in press).

12 Lynn RM, Viner RM, Nicholls DE. Ascertainment of early onset eating disorders: a pilot for developing a national child psychiatric surveillance system. *Child Adolesc Ment Health* 2012;17:109–112.

For peer review only - http://bmjopen.bmj.com/site/about/guidelines.xhtml

For peer review only

13 Knowles RL, Friend H, Lynn R, Mitchell S, Michie C, Ihekweazu C. Surveillance of rare diseases: a public health evaluation of the British Paediatric Surveillance Unit. *J Public Health* 2012;34:279–286.

14 American Psychiatric Association. Diagnostic and Statistical Manual of Mental Disorders (DSM–5), Fifth Edition. Washington DC: American Psychiatric Association, 2013.

15 Le Grange D, Doyle PM, Swanson SA, Ludwig K, Glunz K, Kreipe RE. Calculation of expected body weight in adolescents with eating disorders. *Pediatrics* 2012;129:e438–e446.

16 Gowers SG, Harrington RC, Whitton A, et al. Brief scale for measuring the outcomes of emotional and behavioural disorders in children: Health of the Nation Outcome Scales for Children and Adolescents (HoNOSCA). *Br J Psychiatry* 1999;174:413–416.

17 Shaffer D, Gould MS, Brasic J, et al. A children's global assessment scale (CGAS). *Arch Gen Psychiatry* 1983;40:1228–1231.

18 Office for National Statistics. Population Estimates for UK, England and Wales, Scotland and Northern Ireland. Office for National Statistics, 2015.

19 Central Statistics Office for the Republic of Ireland. PEA11: Population estimates from 1926 by Single Year of Age, Sex and Year, https://www.cso.ie/px/pxeirestat/Statire/SelectVarVal/Define.asp?maintable=PEA11&PLanguage=0.

20 Hakulinen T, Dyba T. Precision of incidence predictions based on Poisson distributed observations. *Stat Med* 1994;13:1513–1523.

21 Gowers S, Levine W, Bailey-Rogers S, Shore A, Burhouse E. Use of a routine, self-report outcome measure (HoNOSCA-SR) in two adolescent mental health services. *Br J Psychiatry* 2002;180:266–269.

22 Gowers SG, Clark A, Roberts C, et al. Clinical effectiveness of treatments for anorexia nervosa in adolescents: Randomised controlled trial. *Br J Psychiatry* 2007;191:427–435.

23 Favaro A, Caregaro L, Tenconi E, Bosello R, Santonastaso P. Time trends in age at onset of anorexia nervosa and bulimia nervosa. *J Clin Psychiatry* 2009;70:1715–1721

24 Reas DL, Rø O. Time trends in healthcare-detected incidence of anorexia nervosa and bulimia nervosa in the Norwegian National Patient Register (2010–2016). *Int J Eat Disord* 2018;51:1144–1152.

For peer review only - http://bmjopen.bmj.com/site/about/guidelines.xhtml

25 NHS England. Access and Waiting Time Standard for Children and Young People with an Eating Disorder: Commissioning Guide. London: National Collaborating Centre for Mental Health, 2015.

For peer review only

For peer review only - http://bmjopen.bmj.com/site/about/guidelines.xhtml

Table 1 Characteristics of incident cases

	N	Mean (SD) or %
Age	305	14.56 (1.66)
Sex		
Female	279	91.48%
Male	26	8.52%
Ethnicity		
Any White	274	91.64%
White and Asian	6	2.01%
White and Black Caribbean	2	0.67%
White and Black African	1	0.33%
Other Mixed	1	0.33%
Indian	3	1.00%
Pakistani	2	0.67%
Bangladeshi	1	0.33%
Other Asian	4	1.34%
Black Caribbean	2	0.67%
Chinese	1	0.33%
Ethnicity not known	2	0.67%
Clinical status		
BMI	304	16.50 (2.25)
% of median expected BMI	303	83.23 (10.99)
CGAS	280	44.61 (14.08)
HoNOSCA	63	19.40 (8.17)

For peer review only

Table 2 Cases by region of the UK and Republic of Ireland

Region	Incident cases N	Incident cases %*	Population N	Population %
England	213	69.84%	6,194,444	77.83%
Scotland	44	14.43%	561,490	7.06%
Northern Ireland	41	13.44%	231,822	2.91%
Republic of Ireland	7	2.30%	628,251	7.89%
Wales	0	0.00%	342,627	4.31%
Total	305		7,958,634	

* Does not sum to 100 due to rounding

For peer review only - http://bmjopen.bmj.com/site/about/guidelines.xhtml

Table 3 Annual incidence of anorexia nervosa in young people aged 8 to 17 for 2015, reported per 100,000 young people

	Observed rate IR0		Adjusted rate IR1		Adjusted rate IR2	
Age	IR	95% CI	IR	95% CI	IR	95% CI
8	0.18	0.01 to 0.76	0.43	0.10 to 1.14	0.57	0.18 to 1.35
9	0.18	0.01 to 0.77	0.44	0.11 to 1.17	0.58	0.18 to 1.38
10	0.19	0.01 to 0.80	0.45	0.11 to 1.21	0.60	0.19 to 1.43
11	1.53	0.79 to 2.67	3.65	2.43 to 5.25	4.85	3.43 to 6.65
12	4.91	3.47 to 6.76	11.69	9.39 to 14.38	15.56	12.89 to 18.63
13	8.39	6.44 to 10.73	19.95	16.89 to 23.42	26.58	23.02 to 30.54
14	11.71	9.41 to 14.39	27.85	24.25 to 31.84	37.10	32.92 to 41.66
15	12.39	10.05 to 15.10	29.47	25.80 to 33.52	39.25	35.50 to 43.88
16	12.76	10.42 to 15.47	30.37	26.70 to 34.41	40.45	36.19 to 45.07
17	6.03	4.47 to 7.96	14.35	11.88 to 17.19	19.12	16.24 to 22.35
Total	5.75	5.23 to 6.30	13.68	12.88 to 14.52	18.22	17.29 to 19.18

For peer review only

For peer review only - http://bmjopen.bmj.com/site/about/guidelines.xhtml

Table 4 Annual incidence of anorexia nervosa in young people aged 8 to 17 for 2015 by sex, reported per 100,000 young people

	Observed incidence IR0				Adjusted incidence IR1				Adjusted incidence IR2			
Age	Female	95% CI	Male	95% CI	Female	95% CI	Male	95% CI	Female	95% CI	Male	95% CI
8	0.36	0.02 to 1.55	0.00	0.00 to 0.00	0.87	0.21 to 2.34	0.00	0.00 to 0.00	1.16	0.36 to 2.76	0.00	0.00 to 0.00
9	0.00	0.00 to 0.00	0.35	0.02 to 1.52	0.00	0.00 to 0.00	0.85	0.21 to 2.28	0.00	0.00 to 0.00	1.13	0.35 to 2.69
10	0.39	0.02 to 1.65	0.00	0.00 to 0.00	0.93	0.23 to 2.48	0.00	0.00 to 0.00	1.23	0.39 to 2.93	0.00	0.00 to 0.00
11	2.35	1.07 to 4.46	0.75	0.15 to 2.18	5.59	3.47 to 8.51	1.77	0.71 to 3.63	7.44	4.96 to 10.72	2.37	1.11 to 4.42
12	8.05	5.43 to 11.50	1.92	0.80 to 3.86	19.17	14.98 to 24.16	4.56	2.69 to 7.22	25.53	20.66 to 31.21	6.09	3.89 to 9.08
13	16.36	12.48 to 21.06	0.78	0.16 to 2.28	38.93	32.81 to 45.86	1.85	0.75 to 3.79	51.83	44.72 to 59.74	2.47	1.16 to 4.62
14	22.35	17.83 to 27.67	1.53	0.56 to 3.32	53.19	46.08 to 61.10	3.64	2.00 to 6.07	70.84	62.58 to 79.88	4.84	2.91 to 7.55
15	24.28	19.59 to 29.74	1.11	0.33 to 2.71	57.77	50.41 to 65.90	2.65	1.31 to 4.78	76.93	68.39 to 86.23	3.54	1.95 to 5.91
16	23.94	19.36 to 29.28	2.16	0.99 to 4.11	56.95	49.75 to 64.90	5.14	3.20 to 7.83	75.87	67.52 to 84.97	6.85	4.57 to 9.87
17	11.27	8.22 to 15.08	1.07	0.32 to 2.60	26.82	21.98 to 32.40	2.54	1.25 to 4.58	35.71	30.09 to 42.07	3.39	1.87 to 5.67
Total	10.78	9.77 to 11.87	0.96	0.68 to 1.30	25.66	24.09 to 27.30	2.28	1.84 to 2.79	34.17	32.36 to 36.06	3.03	2.52 to 3.62

For peer review only

For peer review only - http://bmjopen.bmj.com/site/about/guidelines.xhtml

Figure legends

Figure 1 Flow diagram of case ascertainment

For peer review only

For peer review only - http://bmjopen.bmj.com/site/about/guidelines.xhtml

Web extras

Case notification definition

Please report any child/young person aged 8 to 17 years and 11 months inclusive, who meets the case notification definition criteria below for the first time in the last month. One bullet point criterion from each group below should be fulfilled.

Group A

- Restriction of food, low body weight, or
- Weight less than expected for age

Group B

- Fear of gaining weight, or
- Fear of becoming fat, or
- Behaviour that interferes with weight gain, for example excessive exercising, self-induced vomiting, use of laxatives and diuretics

Group C

- Body image disturbance, or
- Persistent lack of recognition of the seriousness of the current low body weight

Exclusions

- Patients who are not underweight
- Patients with bulimia nervosa, binge eating disorder, avoidant restrictive food intake disorder or other failure to thrive presentations

For peer review only

For peer review only - http://bmjopen.bmj.com/site/about/guidelines.xhtml

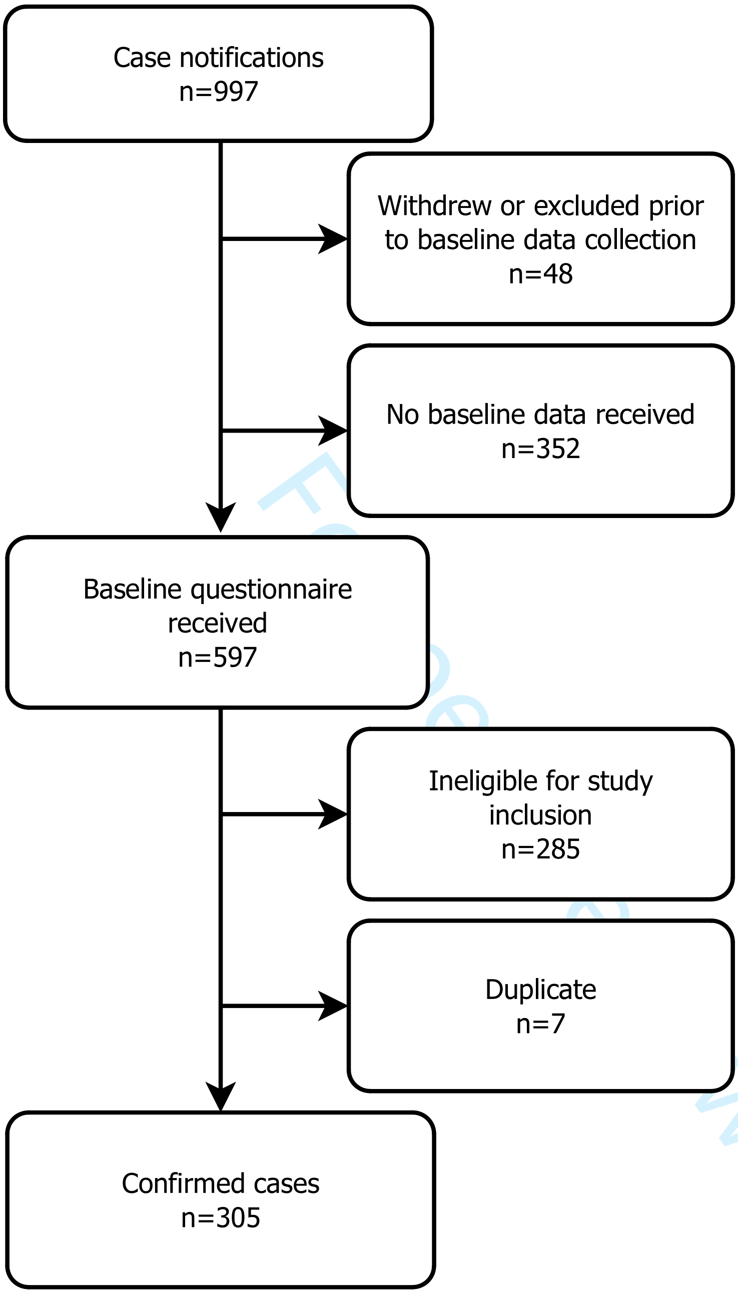
Case notifications
n=997
Withdrew or excluded prior to baseline data collection
n=48
No baseline data received
n=352
Baseline questionnaire received
n=597
Ineligible for study inclusion
n=285
Duplicate
n=7
Confirmed cases
n=305

STROBE Statement—Checklist of items that should be included in reports of ***cross-sectional studies***

	Item No	Recommendation	Page No
**Title and abstract**	1	(*a*) Indicate the study's design with a commonly used term in the title or the abstract	1
		(*b*) Provide in the abstract an informative and balanced summary of what was done and what was found	2
**Introduction**			
Background/rationale	2	Explain the scientific background and rationale for the investigation being reported	5
Objectives	3	State specific objectives, including any prespecified hypotheses	5
**Methods**			
Study design	4	Present key elements of study design early in the paper	5-6
Setting	5	Describe the setting, locations, and relevant dates, including periods of recruitment, exposure, follow-up, and data collection	5 (setting, location), 6 (dates), 7-8 (data)
Participants	6	(*a*) Give the eligibility criteria, and the sources and methods of selection of participants	6 (eligibility), 8 (selection)
Variables	7	Clearly define all outcomes, exposures, predictors, potential confounders, and effect modifiers. Give diagnostic criteria, if applicable	7-8 (outcomes), 8 (diagnostic criteria)
Data sources/ measurement	8*	For each variable of interest, give sources of data and details of methods of assessment (measurement). Describe comparability of assessment methods if there is more than one group	7-8
Bias	9	Describe any efforts to address potential sources of bias	10, 14
Study size	10	Explain how the study size was arrived at	n/a – population level
Quantitative variables	11	Explain how quantitative variables were handled in the analyses. If applicable, describe which groupings were chosen and why	9-11
Statistical methods	12	(*a*) Describe all statistical methods, including those used to control for confounding	11
		(*b*) Describe any methods used to examine subgroups and interactions	n/a
		(*c*) Explain how missing data were addressed	9-11
		(*d*) If applicable, describe analytical methods taking account of sampling strategy	n/a
		(*e*) Describe any sensitivity analyses	n/a
**Results**			
Participants	13*	(a) Report numbers of individuals at each stage of study—eg numbers potentially eligible, examined for eligibility, confirmed eligible, included in the study, completing follow-up, and analysed	11, Figure 1
		(b) Give reasons for non-participation at each stage	11, Figure 1
		(c) Consider use of a flow diagram	Figure 1

For peer review only

For peer review only - http://bmjopen.bmj.com/site/about/guidelines.xhtml

Descriptive data	14*	(a) Give characteristics of study participants (eg demographic, clinical, social) and information on exposures and potential confounders	12, 22 (Table 1)
		(b) Indicate number of participants with missing data for each variable of interest	22 (Table 1)
Outcome data	15*	Report numbers of outcome events or summary measures	n/a
Main results	16	(*a*) Give unadjusted estimates and, if applicable, confounder-adjusted estimates and their precision (eg, 95% confidence interval). Make clear which confounders were adjusted for and why they were included	12-13, 24-25 (Tables 3 & 4)
		(*b*) Report category boundaries when continuous variables were categorized	n/a
		(*c*) If relevant, consider translating estimates of relative risk into absolute risk for a meaningful time period	n/a
Other analyses	17	Report other analyses done—eg analyses of subgroups and interactions, and sensitivity analyses	n/a
**Discussion**			
Key results	18	Summarise key results with reference to study objectives	13
Limitations	19	Discuss limitations of the study, taking into account sources of potential bias or imprecision. Discuss both direction and magnitude of any potential bias	14-15
Interpretation	20	Give a cautious overall interpretation of results considering objectives, limitations, multiplicity of analyses, results from similar studies, and other relevant evidence	15 (interpretation), 13-14 (comparison with similar studies)
Generalisability	21	Discuss the generalisability (external validity) of the study results	14
**Other information**			
Funding	22	Give the source of funding and the role of the funders for the present study and, if applicable, for the original study on which the present article is based	17

*Give information separately for exposed and unexposed groups.

Note: An Explanation and Elaboration article discusses each checklist item and gives methodological background and published examples of transparent reporting. The STROBE checklist is best used in conjunction with this article (freely available on the Web sites of PLoS Medicine at http://www.plosmedicine.org/, Annals of Internal Medicine at http://www.annals.org/, and Epidemiology at http://www.epidem.com/). Information on the STROBE Initiative is available at www.strobe-statement.org.

For peer review only

BMJ Open

Incidence of anorexia nervosa in young people in the United Kingdom and the Republic of Ireland: A national surveillance study

Journal:	*BMJ Open*
Manuscript ID	bmjopen-2018-027339.R2
Article Type:	Research
Date Submitted by the Author:	18-Jul-2019

Complete List of Authors:	Petkova, Hristina; Institute of Psychiatry Psychology and Neuroscience, Health Service and Population Research Department<br>Simic, Mima; South London and Maudsley NHS Foundation Trust<br>Nicholls, Dasha; Imperial College London<br>Ford, Tamsin; University of Exeter<br>Prina, A.Matthew; Institute of Psychiatry Psychology and Neuroscience<br>Stuart, Ruth; Institute of Psychiatry Psychology and Neuroscience, Health Service and Population Research Department<br>Livingstone, Nuala; Queen's University Belfast<br>Kelly, Grace; Queen's University Belfast<br>Macdonald, Geraldine; University of Bristol<br>Eisler, Ivan; South London and Maudsley NHS Foundation Trust<br>Gowers, Simon; University of Liverpool<br>Barrett, Barbara; Institute of Psychiatry Psychology and Neuroscience<br>Byford, Sarah; Institute of Psychiatry Psychology and Neuroscience

**Primary Subject Heading**:	Mental health
Secondary Subject Heading:	Epidemiology
Keywords:	Eating disorders < PSYCHIATRY, Child & adolescent psychiatry < PSYCHIATRY, EPIDEMIOLOGY, anorexia nervosa

SCHOLARONE™
Manuscripts

Incidence of anorexia nervosa in young people in the United Kingdom and the Republic of Ireland: A national surveillance study

Hristina Petkova, Institute of Psychiatry, Psychology & Neuroscience at King's College London (Research Fellow).

Mima Simic, South London and Maudsley NHS Foundation Trust (Consultant Child and Adolescent Psychiatrist).

Dasha Nicholls, Imperial College London (Reader in Child and Adolescent Psychiatry).

Tamsin Ford, University of Exeter Medical School (Professor of Child and Adolescent Psychiatry).

A. Matthew Prina, Institute of Psychiatry, Psychology & Neuroscience at King's College London (Senior Lecturer in Epidemiology).

Ruth Stuart, Institute of Psychiatry, Psychology & Neuroscience at King's College London (Research Assistant).

Nuala Livingstone, Queen's University Belfast (Research Fellow).

Grace Kelly, Queen's University Belfast (Research Fellow).

Geraldine Macdonald, University of Bristol (Professor of Social Work).

Ivan Eisler, South London and Maudsley NHS Foundation Trust (Professor of Family Psychology and Family Therapy).

Simon Gowers, University of Liverpool (Professor of Adolescent Psychiatry).

Barbara Barrett, Institute of Psychiatry, Psychology & Neuroscience at King's College London (Senior Lecturer in Health Economics).

Sarah Byford, Institute of Psychiatry, Psychology & Neuroscience at King's College London (Professor of Health Economics).

Corresponding author: Sarah Byford, Institute of Psychiatry, Psychology & Neuroscience at King's College London, P024 David Goldberg Centre, De Crespigny Park, London SE5 8AF, UK, s.byford@kcl.ac.uk, 02078480043

For peer review only - http://bmjopen.bmj.com/site/about/guidelines.xhtml

Abstract

Objectives: This study aimed to estimate the incidence of DSM5 anorexia nervosa in young people in contact with child and adolescent mental health services in the UK and Republic of Ireland.

Design: Observational, surveillance study using the Child and Adolescent Psychiatry Surveillance System, involving monthly reporting by child and adolescent psychiatrists between 1st February 2015 and 30th September 2015.

Setting: UK and Republic of Ireland

Participants: Clinician-reported data on young people aged 8 to 17 in contact with child and adolescent mental health services for a first episode of anorexia nervosa.

Main outcome measures: Annual incidence rates estimated as confirmed new cases per 100,000 population at risk.

Results: 305 incident cases of anorexia nervosa were reported over the 8-month surveillance period and assessed as eligible for inclusion. The majority were young women (91%), from England (70%), and of white ethnicity (92%). Mean age was 14.6 years (±1.66) and mean percentage of median expected BMI for age and sex was 83.23% (±10.99%). The overall incidence rate, adjusted for missing data, was estimated to be 13.68 per 100,000 population (95% CI 12.88 to 14.52), with rates of 25.66 for young women (95% CI 24.09 to 27.30) and 2.28 for young men (95% CI 1.84 to 2.79). Incidence increased steadily with age, peaking at 15 for young women (57.77, 95% CI 50.41 to 65.90) and 16 for young men (5.14, 95% CI 3.20 to 7.83). Comparison with earlier estimates suggests incidence rates for children aged 13 and under have increased over the last ten years.

Conclusions: These results provide new estimates of the incidence of anorexia nervosa in young people. Service providers and commissioners should consider evidence to suggest an increase in incidence in younger children.

Study registration: International Standard Randomised Controlled Trial Number Register [ISRCTN12676087].

Strengths and limitations of this study

- The study benefits from a large, nationally representative sample from across the UK and the Republic of Ireland.

For peer review only - http://bmjopen.bmj.com/site/about/guidelines.xhtml

For peer review only

- This study used a National surveillance system to collect data and thus avoided biases inherent in studying clinical samples via a small number of centres in a limited number of geographical areas.
- Results were limited by missing data which was dealt with by adjusting observed incidence rates using assumptions about incidence among missing cases.
- Results are relevant to young people diagnosed with anorexia nervosa by child and adolescent psychiatrists and not to those who are managed by general practitioners in primary care or those who have not come to the attention of services, for example those who choose not to seek help.

For peer review only

For peer review only - http://bmjopen.bmj.com/site/about/guidelines.xhtml

Introduction

Anorexia nervosa is a serious and enduring eating disorder with high morbidity and the highest mortality among psychiatric disorders.[1] Young women are particularly susceptible, with annual UK estimates of 37 new diagnoses of anorexia nervosa per 100,000 for girls aged 10 to 19 years, compared to 3 per 100,000 for boys of the same age.[2] Prevalence estimates in young people range from 0.3% to 0.6%.[3–4]

Accurate epidemiological estimates of the number of new anorexia nervosa cases per year and their sex and age profile are needed for causal investigations and service planning.[2] However, available estimates in the UK are at least ten years old.[2,5–7] In addition, most estimates are derived from community-based primary care records,[2,5] which fail to accurately record all new cases.[8,9] Undetected anorexia nervosa cases may present to accident and emergency and require immediate paediatric or psychiatric input, including inpatient admission. Some young people may therefore bypass primary care and, consistent with UK guidelines,[10] are likely to be assessed and diagnosed by a child and adolescent psychiatrist in a secondary care setting, making secondary care records a more reliable source of data on anorexia nervosa incidence than primary care registers.

Existing incidence data from secondary care settings in the UK are limited. One study, focusing only on adolescents aged 13 to 18, was limited to Greater London and reported presentation rates to child and adolescent mental health services (CAMHS), rather than incidence estimates.[6] A second study, which used a national surveillance design, focused only on children under 13.[7] The current study aimed to estimate the incidence of anorexia nervosa in secondary care services for young people between the ages of 8 and 17 years in the UK and the Republic of Ireland. This work formed part of a study exploring the cost-effectiveness of models of care for young people with eating disorders (the CostED study).[11]

Methods

Design

An observational, surveillance study was undertaken using the Child and Adolescent Psychiatry Surveillance System (CAPSS). CAPSS is a system designed to ascertain cases of rare childhood mental health conditions in the UK and Republic of Ireland through monthly reporting by clinicians and relies on non-consent to maximise the accuracy of

For peer review only - http://bmjopen.bmj.com/site/about/guidelines.xhtml

epidemiological estimates. The CAPSS system has been operating since 2009[12] and is based on the well-established British Paediatric Surveillance Unit (BPSU) system.[13]

The study was approved by the CAPSS Executive Committee, King's College London Research Ethics Committee [PNM/13/14-105], and the Health Research Authority [CAG 4-03(PR1)/2014] under Section 251 of the NHS Act 2006, which enables disclosure of confidential patient information for purposes where it is not possible to use anonymised information and where seeking consent is not practical.

Inclusion and exclusion criteria

The study included young people between 8 and 17 years of age, in contact with CAMHS for a first episode of anorexia nervosa according to DSM5 diagnostic criteria.[14] Anorexia nervosa is exceptionally rare in children under 8 and the cut-off at 17 was due to the focus on young people in contact with CAMHS, with many young people transitioning to adult services at the age of 18. New cases were notified for a period of eight months from 1st February to 30th September 2015. Cases whose clinician-reported data were insufficient to assess eligibility were excluded, as were duplicate cases notified more than once by the same or different clinicians.

Procedures

At the time of the study, CAPSS used a report card, known as the yellow card, containing a list of conditions being surveyed. Yellow cards, along with reporting instructions and protocols for new studies, are sent monthly from the CAPSS office to a mailing list of all hospital, university and community child and adolescent consultant psychiatrists across the United Kingdom and the Republic of Ireland. Reporting clinicians are asked to check boxes against any of the reportable conditions they have seen in the preceding month, or to check a "nil return" box and return the card to CAPSS. A tear-off slip is provided for respondents to keep a record of the patients reported. "Positive" returns are allocated a unique CAPSS ID number and notified to the appropriate research investigator, who then contacts the reporting clinician directly to request completion of a questionnaire using the CAPSS ID to enable the clinician to identify the relevant patient.

For the CostED study, the yellow card contained a check box for anorexia nervosa and was sent to clinicians along with a protocol card detailing the case notification definition for

For peer review only - http://bmjopen.bmj.com/site/about/guidelines.xhtml

anorexia nervosa. The case notification definition (see web extras) was based on DSM5 diagnostic criteria for anorexia nervosa and was intended to aid clinicians in their decision to tick "yes" or "no" on the yellow card. It was not intended to identify whether a case met study inclusion criteria, which was determined by the research group after receipt of all necessary data.

Data

Questionnaires were sent to clinicians who reported a positive case of anorexia nervosa, identified via the unique CAPSS ID number. Questionnaires were completed from clinical records and clinicians were asked to provide data relating to the time the case was initially assessed and diagnosed. The questionnaire covered clinical features to enable assessment of case eligibility, referral pathway information to ensure assessment and diagnosis had not happened prior to the study surveillance period, and a limited set of standard patient identifiers in line with CAPSS procedures and ethics requirements, which were used to describe the sample and to identify duplicate notifications. In addition, clinicians were asked to confirm whether the case was a first episode of anorexia nervosa that had come to the attention of services.

The patient identifiers included NHS or Community Health Index (CHI) number (unique patient identifiers used in the regions of interest), hospital number, first half of postcode or town of residence for the Republic of Ireland, sex, date of birth and ethnicity (White, Mixed, Asian, Black, Chinese, Other or Unknown). In Northern Ireland, identifiers were limited to age in years and months and hospital identifier rather than hospital number, to reduce the risk of patient identification given the small geographic area. All patient identifiable data from Northern Ireland were retained by the local research team, de-duplicated, anonymised and subsequently sent to the central research team in King's College London for analysis as per requirements set out by the Northern Ireland Privacy Advisory Committee. All data storage was compliant with the EU General Data Protection Regulations.

Clinical features included: weight and height to calculate body mass index (BMI) and percentage of median expected BMI for age and sex interpreted around the 85% threshold;[15] the Health of the Nation Outcome Scales for Children and Adolescents (HoNOSCA[16]), a routine outcome measure rating 13 clinical features on a five-point severity scale including behaviours, impairments, symptoms, and social functioning of children and adolescents with

For peer review only - http://bmjopen.bmj.com/site/about/guidelines.xhtml

mental health problems; the clinician completed Children's Global Assessment Scale (CGAS[17]) used to rate emotional and behavioural functioning of young people; and a range of symptoms relating to the diagnosis of anorexia nervosa.

Unreturned or incomplete questionnaires were chased via email and telephone. Cases where any symptom required for case definition was absent despite chasing, were assessed for eligibility by a consultant child and adolescent psychiatrist (MS).

Case eligibility

Cases were assessed as eligible for the study if: (a) they were between 8 and 17 years of age; (b) they had no previous episode of anorexia nervosa that had come to the attention of services; (c) they received a clinical assessment in the reporting service during the study surveillance period; (d) they had not been referred from another secondary health service (to ensure assessment and diagnosis had not happened prior to study surveillance period); and (e) the following clinical symptoms were present: "Restriction of energy intake relative to requirements" and "Persistent behaviour that interferes with weight gain, despite low weight". This broad definition was subsequently checked using a tighter DSM5 analytic definition including the following symptoms:

1) "Restriction of energy intake relative to requirements" and
2) "Intense fear of gaining weight or of becoming fat" or "Persistent behaviour that interferes with weight gain, despite low weight" and
3) "Perception that body shape/size is larger than it is" or "Preoccupation with body weight and shape" or "Lack of recognition of the seriousness of the current low body weight"

Only one case which met the broad criteria failed to meet the tighter criteria, thus confirming the validity of the broad criteria.

Removal of duplicates

Duplicates were identified by comparing NHS/CHI numbers, hospital numbers/ hospital identifiers and date of birth/age in years and months, as appropriate. The management of duplicates depended upon the outcome for the original notification for which a duplicate was identified. Four scenarios were considered: (1) duplicates where the original notification met study inclusion criteria were excluded and the original retained; (2) duplicates where the original notification had been excluded because the young person was under 8 years of age or

For peer review only - http://bmjopen.bmj.com/site/about/guidelines.xhtml

did not meet the clinical criteria were assessed as a new case to determine if the case now met eligibility criteria; (3) duplicates where the original notification was excluded due to a previous episode of anorexia nervosa, a diagnosis date prior to the study surveillance period or referral from another secondary care service, were excluded; and (4) duplicates where the original notification contained insufficient information to judge eligibility were checked to see if the duplicate contained the missing information and, if available, the original notification was reassessed for eligibility and the duplicate managed as per the scenarios above.

Data analysis

Data analysis was performed using Stata IC v14.2 and Microsoft Excel 2010. Observed incidence rates (denoted IR0), defined as the number of new cases during a specified period of time in a population at risk for developing the disease, were calculated as follows: the number of confirmed new cases of anorexia nervosa in the 8-month surveillance period converted to 12 months [(N cases over 8 months/8)*12], divided by the population at risk and multiplied by 100,000 to give the rate per 100,000 young people.

*IR0 = (confirmed new cases converted to 12 months)/the population at risk *100,000*

The population at risk was calculated as the total number of children of each year of age and each sex in the UK and Republic of Ireland minus the number of prevalent cases who, once diagnosed, are no longer part of the "at risk" population. Population data for 2015 were obtained from the Office for National Statistics for the UK[18] and the Central Statistics Office for the Republic of Ireland.[19] To estimate the number of prevalent cases each year, incident cases in the previous age band were used as a proxy. For example, incident cases aged eight were used as a proxy for prevalent cases in the estimation of the 'at risk' population aged nine, and so on.

To consider incidence among unobserved missing cases, adjustments were needed for unreturned CAPSS notification cards and questionnaires. For CAPSS notification cards, just over half of all notification cards sent out were returned (50.16%). To account for incidence among the 49.84% of unreturned cards, two assumptions were made, and an appropriate correction applied to IR0, the observed incidence rate:

For peer review only - http://bmjopen.bmj.com/site/about/guidelines.xhtml

Assumption 1: To take into consideration the possibility that unreturned cards are more likely to be 'nil' returns, it was assumed that half of unreturned cards (24.92%) were 'negative' and half followed the same proportion of 'negative' and 'positive' as the returned cards. This assumption translates into a correction coefficient of 1.50 derived from (24.92+50.16)/50.16.

Assumption 2: Making no assumptions of bias in the likelihood of unreturned cards being either positive or negative returns, it was assumed that all unreturned cards followed the same proportion of 'negative' and 'positive' as returned cards. This assumption translates into a correction coefficient of 1.99 derived from (49.84+50.16)/50.16.

These assumptions provide a range of incidence rates, from a minimum (observed incidence rate) to a maximum (assumption 2), within which the actual rate is likely to fall. We hypothesised that assumption 1 provides the most realistic estimate since it assumes a bias in the response rates with greater likelihood that unreturned cards are negative ('nil' returns) but does not assume *all* unreturned cards are 'nil' returns, which is the implicit assumption within IR0.

For unreturned questionnaires, approximately two-thirds of the questionnaires that were sent to clinicians reporting positive cases of anorexia nervosa were returned (63%), leaving one-third (37%) unreturned. Since all these questionnaires relate to a 'positive' notification, we applied a correction coefficient of 1.59 derived from (37+63)/63, which assumes that the incidence rate for the unreturned questionnaires is the same as the incidence rate identified in the returned questionnaires for each year of age.

We then combined the correction coefficients described above, to generate two adjusted incidence rates:

Adjusted incidence rate 1 (IR1) = Confirmed new cases of anorexia nervosa converted to 12 months, multiplied by the correction for unreturned CAPSS notification cards under assumption 1, multiplied by the correction for unreturned questionnaires, then divided by the population at risk and multiplied by 100,000**.**

*IR1 = (confirmed new cases converted to 12 months * 1.50 * 1.59)/the population at risk *100,000*

For peer review only - http://bmjopen.bmj.com/site/about/guidelines.xhtml

For peer review only

Adjusted Incidence rate 2 (IR2) = Confirmed new cases of anorexia nervosa converted to 12-months, multiplied by the correction for unreturned CAPSS notification cards under assumption 2, multiplied by the correction for unreturned questionnaires and then divided by the population at risk and multiplied by 100,000.

*IR2 = (confirmed new cases converted to 12 months * 1.99 * 1.59)/the population at risk * 100,000*

For each incidence rate, IR0, IR1 and IR2, total, age-specific and sex-specific annual incidence rates for anorexia nervosa for the year 2015 and 95% confidence intervals were calculated based on the Poisson distribution[20] using the Stata command *ci means [N new anorexia nervosa cases 12m], Poisson [exposure(total population)]* for positive integers/whole incidence numbers (Stata interprets any non-integer decimal point number between 0 and 1 as the fraction of events and converts it to an integer number). Annual incidence rates were stratified by discrete age and sex.

Public and patient involvement statement

The CostED study included a patient and a parent representative on the study steering committee who contributed to the design, conduct and management of the study, including the incidence component.

Results

Case ascertainment

Case ascertainment is outlined in Figure 1. Over the eight-month surveillance period, 6401 yellow cards were sent to reporting clinicians and 3211 were returned (50%). Of these, 997 positive cases of anorexia nervosa were reported and 2214 were nil returns. Of the positive cases, 48 (5%) were excluded due to clinicans stating that they did not wish to be included in the study (due to retirement, shortage of reporting capacity etc.) or due to reporting errors. Questionnaires were sent to the remaining 949, and a further 352 positive cases (37%) were excluded as they failed to return the questionnaires, so no data were available to assess eligibility. Questionnaires were completed and returned for 597 notified cases, of which 292 (49%) were ineligible for reasons related to age, previous episode of anorexia nervosa, date of assessment outside the study's surveillance period, referral from another secondary care

For peer review only - http://bmjopen.bmj.com/site/about/guidelines.xhtml

service, insufficient information to assess diagnosis or duplicate notifications, leaving 305 incident cases of anorexia nervosa as the sample for analysis.

Demographics and clinical features

Of the 305 young people identified as having DSM5 anorexia nervosa, the majority were girls (91%), from England (70%) and of white ethnicity (92%) (see Table 1). The mean age was 14.6 years (±1.66). Clinical variables suggest these young people were significantly impaired. Mean BMI was 16.50 kg/m^2 (±2.25), where values of 16.00 to 16.99 suggesting moderate severity of anorexia nervosa. Mean percentage of median expected BMI for age and sex (the deviation from expected body weight) was 83.23% (±10.99%), falling within the range required for a diagnosis of anorexia nervosa (<85%). Mean CGAS score was 44.61 (±14.08), which falls within the range for 'obvious problems' (41–50) on a scale from 1 to 100 (1 being the worst and 100 the best emotional and behavioural functioning). Mean total HoNOSCA score was 19.40 (±8.17) on a scale from 0 to 52, indicative of a severity similar to that at inpatient admission.[21,22]

The proportion of the included sample notified from each region within the British Isles is reported in Table 2, alongside the population of young people in each region by age. England has the largest population (78%) and notified 70% of new cases. Scotland, containing only 7% of the total population, notified 14% of the sample and Northern Ireland, containing only 3% of the population, notified 13% of the sample. By contrast, the Republic of Ireland notified only 2% of cases, despite containing 8% of the population, and Wales notified no eligible cases (some cases were notified but did not meet inclusion criteria), despite containing 4% of the population.

Incidence rates

Table 3 details observed incidence rates (IR0) and adjusted incidence rates (IR1 and IR2) by age. Incidence rates for the total sample ranged from a minimum of 5.75 per 100,000 young people (95% CI 5.23 to 6.30; IR0) to a maximum of 18.22 per 100,000 young people (95% CI 17.29 to 19.18; IR2), with IR1, the rate hypothesised to be the most accurate, falling between these two values at 13.68 per 100,000 population (95% CI 12.88 to 14.52). Focusing on IR1 rates, total incidence increased steadily with age, peaking at 16 (30.37, 95% CI 26.70 to 34.41), with a substantial drop at the age of 17 (14.35, 95% CI 11.88 to 17.19).

For peer review only - http://bmjopen.bmj.com/site/about/guidelines.xhtml

Table 4 reports incidence rates by age and sex. Incidence among young men followed a similar pattern to overall incidence rates reported in Table 3, being highest at the age of 16 (5.14) and half that at age 17 (2.54). The highest incidence among young women was seen a year earlier than for boys, at the age of 15 (57.77), with similar rates at age 16 (56.95), dropping by more than half at age 17 (26.82).

Discussion

Principal findings

This study provides new estimates of incident cases of anorexia nervosa in young people aged 8 to 17 presenting to CAMHS services in the UK and Republic of Ireland. Our mid-range, missing data-adjusted estimate (IR1) of the incidence of anorexia nervosa in the full sample of young people aged 8 to 17 years was approximately 14 per 100,000.

Comparison with other studies

This result is lower than previous primary care-based estimates of 18–20 per 100,000 focusing on young people aged 10–19.[2,5] This difference is due to the different age ranges in the studies; the inclusion of children as young as 8 in the current study, who have relatively low incidence, and exclusion of adolescents aged 18 to 19, whose incidence is relatively high, makes the results difficult to compare. However, comparing rates for 10 to 14-year olds, available in both studies, produces similar incidence rates, with rates of 12.6 per 100,000 in the current study, compared to 13.1 per 100,000 in 2009.[2] For females, the rates are 23.3 per 100,000 in the current study compared to 24.0 per 100,000 in 2009 and for males, 2.4 and 2.5 per 100,000, respectively. However, this comparison should be treated with caution given the very different settings – primary care versus secondary care.

Existing secondary care estimates of the incidence of anorexia nervosa in the UK are limited to children under the age of 13, with an overall incidence of 1.09 per 100,000 reported for children aged between 6 and 13 between 2005 and 2006.[7] The methodology for this study was very similar to the CostED methodology, using the CAPSS system but additionally the British Paediatric Surveillance System. For comparison with the current study, the incidence rate for children between 8 and 13 was approximately 1.5 per 100,000 for DSMIV anorexia nervosa or 1.8 per 100,000 for DSMIV anorexia nervosa plus 'other eating disorders' likely to contain cases that would now be classified as anorexia nervosa using DSM5. This compares to a rate of 5.83 per 100,000 in the current study for children of the same age. This estimate is

For peer review only - http://bmjopen.bmj.com/site/about/guidelines.xhtml

substantially higher than the 2006 estimates suggesting that incidence rates for younger children have increased over time.

The results presented are also supported by international evidence. One study carried out in Italy demonstrated a significant reduction in age at onset for anorexia nervosa in consecutive outpatient referrals between 1985 and 2008 (n=1,666).[23] A second study exploring time trends in the incidence of anorexia nervosa, which was carried out using data from the Norwegian National Patient Register, found overall rates of anorexia nervosa to be stable between 2010 and 2016 for the sample as a whole, but increasing for young females aged between 10 and 14.[24]

Strengths and weaknesses of the study

The large, nationally representative sample of this study is a strength. The study included young people with anorexia nervosa, diagnosed using DSM5 criteria, from across the UK and the Republic of Ireland and thus avoided biases inherent in studying clinical samples via a small number of centres in a limited number of geographical areas. The results are of relevance primarily to the UK and Republic of Ireland but may be of value to other high-income countries.

With only a 50% response rate from CAPSS clinicians and a third of questionnaires not returned, missing data were a major constraint. There are many reasons why clinicians may fail to return notification cards or questionnaires, including changes in place of employment, competing priorities, or the belief that cases will be reported by a colleague.[13] This problem was addressed by adjusting the observed incidence rates using assumptions about incidence among both missing case notifications and missing questionnaires.

The methodology is also limited to young people seen by child and adolescent psychiatrists. Cases that would not be identified by this methodology include those who have not come to the attention of services, for example those who choose not to seek help, those managed by general practitioners in primary care, and those in the care of mental health services without psychiatric input, such as nurse-led facilities. This latter concern was an issue in Northern Ireland where, due to initial low numbers of notifications, investigation by the research team identified a number of nurse-led facilities which were invited to contribute, and subsequently reported just over half of all cases in Northern Ireland. In terms of missing primary care

For peer review only - http://bmjopen.bmj.com/site/about/guidelines.xhtml

cases, given UK guidelines for assessment and diagnosis of anorexia nervosa to be carried out by child and adolescent psychiatrists in secondary care settings,[10] it is reasonable to assume that many of those cases remaining in primary care would not meet criteria for DSM5 anorexia nervosa. It is also possible that current inpatient cases are under-represented; although notifications were sent to all child and adolescent psychiatrists, including those working in inpatient settings, the main focus of the CostED study was the evaluation of community-based services, and so clinicians may have mistakenly focused on notification of community-based cases.

It must also be borne in mind that service-level (rather than population-level) incidence rates are sensitive to external factors, including service availability, funding and commissioning decisions, parental and school awareness, stigma, etc., all of which will impact upon observed trends in incidence rates over time. The nature of community-based eating disorders services for children and adolescents in England has started to change following the publication of commissioning standards in June 2015,[25] as well as investment of £30 million to support the development of these services. The CostED incidence data were collected in 2015, one year before the first allocation of funding to services was made in 2016, and thus these initiatives, which may result in increases in observed incidence rates in the future, are not reflected in the data presented. Nevertheless, these estimates are approximately ten years more recent than existing secondary care data for the UK (collected between 2005 and 2006)[7] and cover a wider age range.

Unanswered questions and future research

Future research should explore the development of earlier interventions, given evidence of an increase in incidence in young children suggesting that onset of anorexia nervosa may be starting earlier for some young people than suggested by previous research. Research is also needed to identify approaches to the assessment of incidence simultaneously in primary and secondary care. Multinational studies should be considered for better assessment and exploration of incidence rates in young men.

Conclusion

These results provide new estimates of the incidence of anorexia nervosa in young people in the UK and Republic of Ireland. Whilst firm conclusions relating to changes in incidence rates over time for the entire sample cannot be drawn due to lack of existing secondary care

For peer review only - http://bmjopen.bmj.com/site/about/guidelines.xhtml

evidence, service providers and commissioners should consider evidence to suggest an increase in incidence in younger children.

Word count

4111

Acknowledgements

We would like to thank all the clinicians who were kind enough to give up their time to provide the CostED study with CAPSS notifications and to complete questionnaires. We know your time is precious and in short supply and we are truly grateful. We would also like to thank the team at the Child and Adolescent Psychiatry Surveillance System, based at the Royal College of Psychiatrist's Centre for Care Quality and Improvement, for their support with the design and running of the surveillance aspect of this study, and the study steering committee for their invaluable advice.

Contributor and guarantor information

Hristina Petkova contributed to data collection, data entry, data cleaning, data analysis, and drafting of the manuscript.

Mima Simic was a co-applicant, contributed to the design of the study, provided clinical expertise, and commented on and approved the manuscript.

Dasha Nicholls was a co-applicant, contributed to the design of the study, provided expertise on CAPSS methodology, clinical expertise, and commented on and approved the manuscript.

Tamsin Ford was a co-applicant, contributed to the design of the study, provided clinical expertise, and commented on and approved the manuscript.

Matthew Prina contributed to the data analysis and commented on and approved the manuscript.

Ruth Stuart contributed to data collection, data entry, data cleaning and data analysis, and commented on and approved the manuscript.

Nuala Livingstone contributed to data collection, data entry and data management in Northern Ireland, and commented on and approved the manuscript.

Grace Kelly contributed to data collection, data entry and data management in Northern Ireland, and commented on and approved the manuscript.

Geraldine Macdonald contributed to the design of the study, managed the Northern Ireland research staff, and commented on and approved the final report.

For peer review only - http://bmjopen.bmj.com/site/about/guidelines.xhtml

Ivan Eisler was a co-applicant, contributed to the design of the study, provided clinical support to the research team, and commented on and approved the manuscript.

Simon Gowers was a co-applicant, contributed to the design of the study, provided clinical expertise, and commented on and approved the manuscript.

Barbara Barrett was a co-applicant, contributed to the design of the study, the data analysis, and commented on and approved the manuscript.

Sarah Byford was principal investigator, led the study, managed the King's College London research staff, contributed to the design of the study and the data analysis, drafted the paper, and is responsible for the overall content as guarantor.

Competing interests

Tamsin Ford reports she is Chair of the Child and Adolescent Psychiatry Surveillance Service that was used to run part of the study, which is an unpaid position (other than travel expenses). Kandarp Joshi reports that he was principal investigator for the Aberdeen site for a Sunovion sponsored multisite trial on effectiveness of Lurasidone in paediatric schizophrenia. No other authors report conflicts of interest.

Transparency statement

The lead author affirms that this manuscript is an honest, accurate, and transparent account of the study being reported; that no important aspects of the study have been omitted; and that any discrepancies from the study as planned and registered have been explained.

Role of the funding source

The study was funded by the NIHR Health Service & Delivery Research programme (11/1023/17). The study funders had no role in the study design, the collection, analysis or interpretation of data, the writing of the report, or the decision to submit the article for publication. The authors were independent from the funders and all authors had full access to all of the data in the study and can take responsibility for the integrity of the data and the accuracy of the data analysis.

Disclaimer

The views expressed are those of the authors and not necessarily those of the NHS, the NIHR or the Department of Health and Social Care.

For peer review only

Data sharing

As a result of the collection of confidential patient data without consent, and approval from the Health Research Authority (following advice from the Confidentiality Advisory Group) for data to be provided for the purposes of the specified activity only, the data cannot be made publicly available for other purposes. However, the CostED research group will consider requests for further analysis on a case by case basis, subject to appropriate ethical/HRA approvals. All data enquiries should be submitted to the corresponding author for consideration in the first instance.

For peer review only

For peer review only - http://bmjopen.bmj.com/site/about/guidelines.xhtml

References

1 Arcelus J, Mitchell AJ, Wales J, Nielsen S. Mortality rates in patients with anorexia nervosa and other eating disorders: A meta-analysis of 36 Studies. *Arch Gen Psychiatry* 2011;68:724–731.

2 Micali N, Hagberg KW, Petersen I, Treasure JL. The incidence of eating disorders in the UK in 2000-2009: findings from the General Practice Research Database. *BMJ Open* 2013;3:e002646.

3 Hoek HW. Incidence, prevalence and mortality of anorexia nervosa and other eating disorders. *Curr Opin Psychiatry* 2006;19:389–394.

4 Smink FRE, van Hoeken D, Hoek HW. Epidemiology of eating disorders: incidence, prevalence and mortality rates. *Current Psychiatry Reports* 2012;14:406–414.

5 Currin L, Schmidt U, Treasure J, Jick H. Time trends in eating disorder incidence. *Br J Psychiatry* 2005;186:132–135.

6 House J, Schmidt U, Craig M, et al. Comparison of specialist and nonspecialist care pathways for adolescents with anorexia nervosa and related eating disorders. *Int J Eat Disord* 2012;45:949–956.

7 Nicholls DE, Lynn R, Viner RM. Childhood eating disorders: British national surveillance study. *Br J Psychiatry* 2011;98:295–301.

8 Lask B, Bryant-Waugh R, Wright F, Campbell M, Willoughby K, Waller G. Family physician consultation patterns indicate high risk for early-onset anorexia nervosa. *Int J Eat Disord* 2005;38:269–272.

9 Goodman R, Ford T, Simmons H, Gatward R, Meltzer H. Using the Strengths and Difficulties Questionnaire (SDQ) to screen for child psychiatric disorders in a community sample. *Br J Psychiatry* 2000;177:534–539.

10 National Institute for Health and Care Excellence. Eating disorders: recognition and treatment. NICE guideline [NG69]. National Institute for Health and Care Excellence, 2017.

11 Byford S, Petkova H, Stuart R, et al. Alternative community-based models of care for young people with anorexia nervosa: the CostED national surveillance study. *Health Service and Delivery Research* 2019 (in press).

12 Lynn RM, Viner RM, Nicholls DE. Ascertainment of early onset eating disorders: a pilot for developing a national child psychiatric surveillance system. *Child Adolesc Ment Health* 2012;17:109–112.

For peer review only - http://bmjopen.bmj.com/site/about/guidelines.xhtml

13 Knowles RL, Friend H, Lynn R, Mitchell S, Michie C, Ihekweazu C. Surveillance of rare diseases: a public health evaluation of the British Paediatric Surveillance Unit. *J Public Health* 2012;34:279–286.

14 American Psychiatric Association. Diagnostic and Statistical Manual of Mental Disorders (DSM–5), Fifth Edition. Washington DC: American Psychiatric Association, 2013.

15 Le Grange D, Doyle PM, Swanson SA, Ludwig K, Glunz K, Kreipe RE. Calculation of expected body weight in adolescents with eating disorders. *Pediatrics* 2012;129:e438–e446.

16 Gowers SG, Harrington RC, Whitton A, et al. Brief scale for measuring the outcomes of emotional and behavioural disorders in children: Health of the Nation Outcome Scales for Children and Adolescents (HoNOSCA). *Br J Psychiatry* 1999;174:413–416.

17 Shaffer D, Gould MS, Brasic J, et al. A children's global assessment scale (CGAS). *Arch Gen Psychiatry* 1983;40:1228–1231.

18 Office for National Statistics. Population Estimates for UK, England and Wales, Scotland and Northern Ireland. Office for National Statistics, 2015.

19 Central Statistics Office for the Republic of Ireland. PEA11: Population estimates from 1926 by Single Year of Age, Sex and Year, https://www.cso.ie/px/pxeirestat/Statire/SelectVarVal/Define.asp?maintable=PEA11&PLanguage=0.

20 Hakulinen T, Dyba T. Precision of incidence predictions based on Poisson distributed observations. *Stat Med* 1994;13:1513–1523.

21 Gowers S, Levine W, Bailey-Rogers S, Shore A, Burhouse E. Use of a routine, self-report outcome measure (HoNOSCA-SR) in two adolescent mental health services. *Br J Psychiatry* 2002;180:266–269.

22 Gowers SG, Clark A, Roberts C, et al. Clinical effectiveness of treatments for anorexia nervosa in adolescents: Randomised controlled trial. *Br J Psychiatry* 2007;191:427–435.

23 Favaro A, Caregaro L, Tenconi E, Bosello R, Santonastaso P. Time trends in age at onset of anorexia nervosa and bulimia nervosa. *J Clin Psychiatry* 2009;70:1715–1721

24 Reas DL, Rø O. Time trends in healthcare-detected incidence of anorexia nervosa and bulimia nervosa in the Norwegian National Patient Register (2010–2016). *Int J Eat Disord* 2018;51:1144–1152.

For peer review only - http://bmjopen.bmj.com/site/about/guidelines.xhtml

For peer review only

25 NHS England. Access and Waiting Time Standard for Children and Young People with an Eating Disorder: Commissioning Guide. London: National Collaborating Centre for Mental Health, 2015.

For peer review only

For peer review only - http://bmjopen.bmj.com/site/about/guidelines.xhtml

Table 1 Characteristics of incident cases

	N	Mean (SD) or %
Age	305	14.56 (1.66)
Sex		
Female	279	91.48%
Male	26	8.52%
Ethnicity		
Any White	274	91.64%
White and Asian	6	2.01%
White and Black Caribbean	2	0.67%
White and Black African	1	0.33%
Other Mixed	1	0.33%
Indian	3	1.00%
Pakistani	2	0.67%
Bangladeshi	1	0.33%
Other Asian	4	1.34%
Black Caribbean	2	0.67%
Chinese	1	0.33%
Ethnicity not known	2	0.67%
Clinical status		
BMI	304	16.50 (2.25)
% of median expected BMI	303	83.23 (10.99)
CGAS	280	44.61 (14.08)
HoNOSCA	63	19.40 (8.17)

For peer review only

For peer review only - http://bmjopen.bmj.com/site/about/guidelines.xhtml

Table 2 Cases by region of the UK and Republic of Ireland

Region	Incident cases N	Incident cases %*	Population N	Population %
England	213	69.84%	6,194,444	77.83%
Scotland	44	14.43%	561,490	7.06%
Northern Ireland	41	13.44%	231,822	2.91%
Republic of Ireland	7	2.30%	628,251	7.89%
Wales	0	0.00%	342,627	4.31%
Total	305		7,958,634	

* Does not sum to 100 due to rounding

For peer review only

For peer review only - http://bmjopen.bmj.com/site/about/guidelines.xhtml

Table 3 Annual incidence of anorexia nervosa in young people aged 8 to 17 for 2015, reported per 100,000 young people

	Observed rate IR0		Adjusted rate IR1		Adjusted rate IR2	
Age	IR	95% CI	IR	95% CI	IR	95% CI
8	0.18	0.01 to 0.76	0.43	0.10 to 1.14	0.57	0.18 to 1.35
9	0.18	0.01 to 0.77	0.44	0.11 to 1.17	0.58	0.18 to 1.38
10	0.19	0.01 to 0.80	0.45	0.11 to 1.21	0.60	0.19 to 1.43
11	1.53	0.79 to 2.67	3.65	2.43 to 5.25	4.85	3.43 to 6.65
12	4.91	3.47 to 6.76	11.69	9.39 to 14.38	15.56	12.89 to 18.63
13	8.39	6.44 to 10.73	19.95	16.89 to 23.42	26.58	23.02 to 30.54
14	11.71	9.41 to 14.39	27.85	24.25 to 31.84	37.10	32.92 to 41.66
15	12.39	10.05 to 15.10	29.47	25.80 to 33.52	39.25	35.50 to 43.88
16	12.76	10.42 to 15.47	30.37	26.70 to 34.41	40.45	36.19 to 45.07
17	6.03	4.47 to 7.96	14.35	11.88 to 17.19	19.12	16.24 to 22.35
Total	5.75	5.23 to 6.30	13.68	12.88 to 14.52	18.22	17.29 to 19.18

For peer review only

Table 4 Annual incidence of anorexia nervosa in young people aged 8 to 17 for 2015 by sex, reported per 100,000 young people

	Observed incidence IR0				Adjusted incidence IR1				Adjusted incidence IR2			
Age	Female	95% CI	Male	95% CI	Female	95% CI	Male	95% CI	Female	95% CI	Male	95% CI
8	0.36	0.02 to 1.55	0.00	0.00 to 0.00	0.87	0.21 to 2.34	0.00	0.00 to 0.00	1.16	0.36 to 2.76	0.00	0.00 to 0.00
9	0.00	0.00 to 0.00	0.35	0.02 to 1.52	0.00	0.00 to 0.00	0.85	0.21 to 2.28	0.00	0.00 to 0.00	1.13	0.35 to 2.69
10	0.39	0.02 to 1.65	0.00	0.00 to 0.00	0.93	0.23 to 2.48	0.00	0.00 to 0.00	1.23	0.39 to 2.93	0.00	0.00 to 0.00
11	2.35	1.07 to 4.46	0.75	0.15 to 2.18	5.59	3.47 to 8.51	1.77	0.71 to 3.63	7.44	4.96 to 10.72	2.37	1.11 to 4.42
12	8.05	5.43 to 11.50	1.92	0.80 to 3.86	19.17	14.98 to 24.16	4.56	2.69 to 7.22	25.53	20.66 to 31.21	6.09	3.89 to 9.08
13	16.36	12.48 to 21.06	0.78	0.16 to 2.28	38.93	32.81 to 45.86	1.85	0.75 to 3.79	51.83	44.72 to 59.74	2.47	1.16 to 4.62
14	22.35	17.83 to 27.67	1.53	0.56 to 3.32	53.19	46.08 to 61.10	3.64	2.00 to 6.07	70.84	62.58 to 79.88	4.84	2.91 to 7.55
15	24.28	19.59 to 29.74	1.11	0.33 to 2.71	57.77	50.41 to 65.90	2.65	1.31 to 4.78	76.93	68.39 to 86.23	3.54	1.95 to 5.91
16	23.94	19.36 to 29.28	2.16	0.99 to 4.11	56.95	49.75 to 64.90	5.14	3.20 to 7.83	75.87	67.52 to 84.97	6.85	4.57 to 9.87
17	11.27	8.22 to 15.08	1.07	0.32 to 2.60	26.82	21.98 to 32.40	2.54	1.25 to 4.58	35.71	30.09 to 42.07	3.39	1.87 to 5.67
Total	10.78	9.77 to 11.87	0.96	0.68 to 1.30	25.66	24.09 to 27.30	2.28	1.84 to 2.79	34.17	32.36 to 36.06	3.03	2.52 to 3.62

For peer review only

Figure legends

Figure 1 Flow diagram of case ascertainment

For peer review only

For peer review only - http://bmjopen.bmj.com/site/about/guidelines.xhtml

Web extras

Case notification definition

Please report any child/young person aged 8 to 17 years and 11 months inclusive, who meets the case notification definition criteria below for the first time in the last month. One bullet point criterion from each group below should be fulfilled.

Group A

- Restriction of food, low body weight, or
- Weight less than expected for age

Group B

- Fear of gaining weight, or
- Fear of becoming fat, or
- Behaviour that interferes with weight gain, for example excessive exercising, self-induced vomiting, use of laxatives and diuretics

Group C

- Body image disturbance, or
- Persistent lack of recognition of the seriousness of the current low body weight

Exclusions

- Patients who are not underweight
- Patients with bulimia nervosa, binge eating disorder, avoidant restrictive food intake disorder or other failure to thrive presentations

For peer review only

For peer review only - http://bmjopen.bmj.com/site/about/guidelines.xhtml

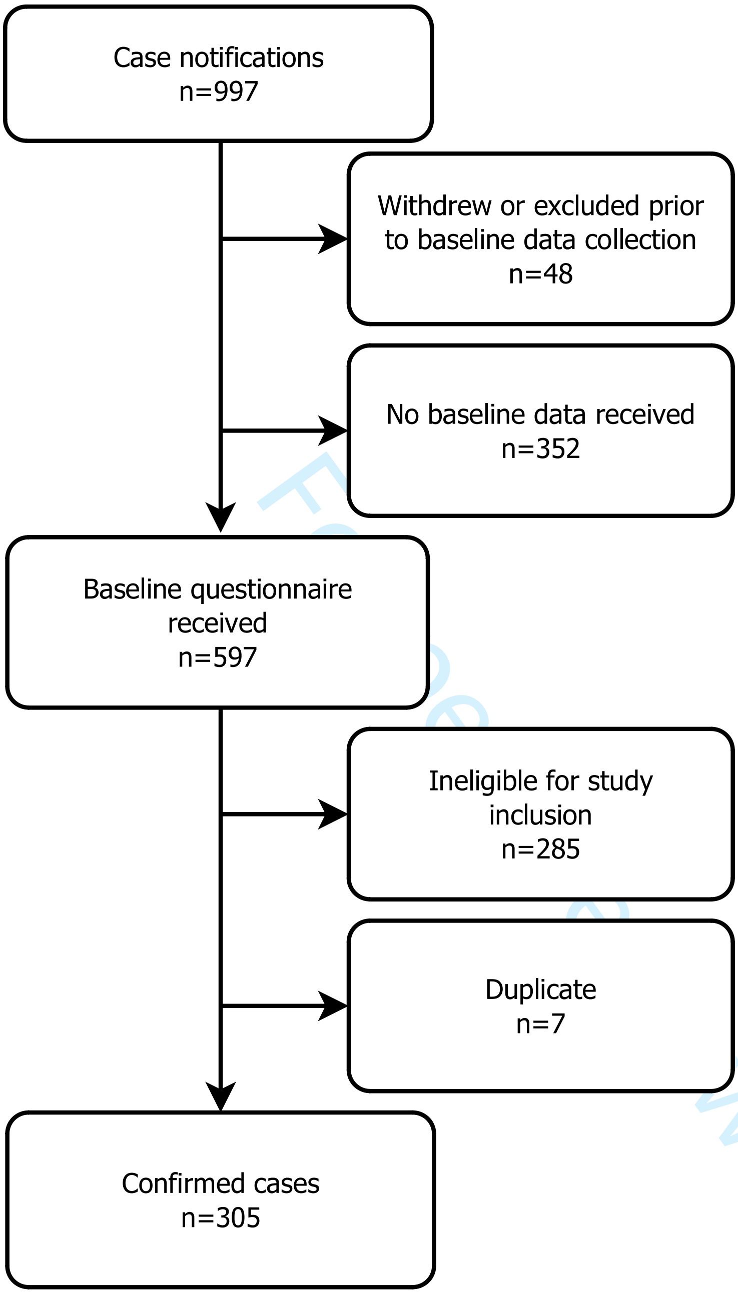

For peer review only

STROBE Statement—Checklist of items that should be included in reports of ***cross-sectional studies***

	Item No	Recommendation	Page No
**Title and abstract**	1	(*a*) Indicate the study's design with a commonly used term in the title or the abstract	1
		(*b*) Provide in the abstract an informative and balanced summary of what was done and what was found	2
**Introduction**			
Background/rationale	2	Explain the scientific background and rationale for the investigation being reported	5
Objectives	3	State specific objectives, including any prespecified hypotheses	5
**Methods**			
Study design	4	Present key elements of study design early in the paper	5-6
Setting	5	Describe the setting, locations, and relevant dates, including periods of recruitment, exposure, follow-up, and data collection	5 (setting, location), 6 (dates), 7-8 (data)
Participants	6	(*a*) Give the eligibility criteria, and the sources and methods of selection of participants	6 (eligibility), 8 (selection)
Variables	7	Clearly define all outcomes, exposures, predictors, potential confounders, and effect modifiers. Give diagnostic criteria, if applicable	7-8 (outcomes), 8 (diagnostic criteria)
Data sources/ measurement	8*	For each variable of interest, give sources of data and details of methods of assessment (measurement). Describe comparability of assessment methods if there is more than one group	7-8
Bias	9	Describe any efforts to address potential sources of bias	10, 14
Study size	10	Explain how the study size was arrived at	n/a – population level
Quantitative variables	11	Explain how quantitative variables were handled in the analyses. If applicable, describe which groupings were chosen and why	9-11
Statistical methods	12	(*a*) Describe all statistical methods, including those used to control for confounding	11
		(*b*) Describe any methods used to examine subgroups and interactions	n/a
		(*c*) Explain how missing data were addressed	9-11
		(*d*) If applicable, describe analytical methods taking account of sampling strategy	n/a
		(*e*) Describe any sensitivity analyses	n/a
**Results**			
Participants	13*	(a) Report numbers of individuals at each stage of study—eg numbers potentially eligible, examined for eligibility, confirmed eligible, included in the study, completing follow-up, and analysed	11, Figure 1
		(b) Give reasons for non-participation at each stage	11, Figure 1
		(c) Consider use of a flow diagram	Figure 1

For peer review only

For peer review only - http://bmjopen.bmj.com/site/about/guidelines.xhtml

Descriptive data	14*	(a) Give characteristics of study participants (eg demographic, clinical, social) and information on exposures and potential confounders	12, 22 (Table 1)
		(b) Indicate number of participants with missing data for each variable of interest	22 (Table 1)
Outcome data	15*	Report numbers of outcome events or summary measures	n/a
Main results	16	(*a*) Give unadjusted estimates and, if applicable, confounder-adjusted estimates and their precision (eg, 95% confidence interval). Make clear which confounders were adjusted for and why they were included	12-13, 24-25 (Tables 3 & 4)
		(*b*) Report category boundaries when continuous variables were categorized	n/a
		(*c*) If relevant, consider translating estimates of relative risk into absolute risk for a meaningful time period	n/a
Other analyses	17	Report other analyses done—eg analyses of subgroups and interactions, and sensitivity analyses	n/a
**Discussion**			
Key results	18	Summarise key results with reference to study objectives	13
Limitations	19	Discuss limitations of the study, taking into account sources of potential bias or imprecision. Discuss both direction and magnitude of any potential bias	14-15
Interpretation	20	Give a cautious overall interpretation of results considering objectives, limitations, multiplicity of analyses, results from similar studies, and other relevant evidence	15 (interpretation), 13-14 (comparison with similar studies)
Generalisability	21	Discuss the generalisability (external validity) of the study results	14
**Other information**			
Funding	22	Give the source of funding and the role of the funders for the present study and, if applicable, for the original study on which the present article is based	17

*Give information separately for exposed and unexposed groups.

Note: An Explanation and Elaboration article discusses each checklist item and gives methodological background and published examples of transparent reporting. The STROBE checklist is best used in conjunction with this article (freely available on the Web sites of PLoS Medicine at http://www.plosmedicine.org/, Annals of Internal Medicine at http://www.annals.org/, and Epidemiology at http://www.epidem.com/). Information on the STROBE Initiative is available at www.strobe-statement.org.

For peer review only
